# Members of the autophagy class III phosphatidylinositol 3-kinase complex I interact with GABARAP and GABARAPL1 via LIR motifs

**DOI:** 10.1080/15548627.2019.1581009

**Published:** 2019-03-04

**Authors:** Åsa Birna Birgisdottir, Stephane Mouilleron, Zambarlal Bhujabal, Martina Wirth, Eva Sjøttem, Gry Evjen, Wenxin Zhang, Rebecca Lee, Nicola O’Reilly, Sharon A. Tooze, Trond Lamark, Terje Johansen

**Affiliations:** a Molecular Cancer Research Group, Department of Medical Biology, University of Tromsø –The Arctic University of Norway, Tromsø, Norway; b Structural Biology, The Francis Crick Institute, London, UK; c Molecular Cell Biology of Autophagy Laboratory, The Francis Crick Institute, London, UK; d Peptide Chemistry Science Technology Platform, The Francis Crick Institute, London, UK

**Keywords:** Autophagy, ATG14, BECN1, GABARAP, LIR, PIK3C3

## Abstract

Autophagosome formation depends on a carefully orchestrated interplay between membrane-associated protein complexes. Initiation of macroautophagy/autophagy is mediated by the ULK1 (unc-51 like autophagy activating kinase 1) protein kinase complex and the autophagy-specific class III phosphatidylinositol 3-kinase complex I (PtdIns3K-C1). The latter contains PIK3C3/VPS34, PIK3R4/VPS15, BECN1/Beclin 1 and ATG14 and phosphorylates phosphatidylinositol to generate phosphatidylinositol 3-phosphate (PtdIns3P). Here, we show that PIK3C3, BECN1 and ATG14 contain functional LIR motifs and interact with the Atg8-family proteins with a preference for GABARAP and GABARAPL1. High resolution crystal structures of the functional LIR motifs of these core components of PtdIns3K-C1were obtained. Variation in hydrophobic pocket 2 (HP2) may explain the specificity for the GABARAP family. Mutation of the LIR motif in ATG14 did not prevent formation of the PtdIns3K-C1 complex, but blocked colocalization with MAP1LC3B/LC3B and impaired mitophagy. The ULK-mediated phosphorylation of S29 in ATG14 was strongly dependent on a functional LIR motif in ATG14. GABARAP-preferring LIR motifs in PIK3C3, BECN1 and ATG14 may, via coincidence detection, contribute to scaffolding of PtdIns3K-C1 on membranes for efficient autophagosome formation.

**Abbreviations:** ATG: autophagy-related; BafA1: bafilomycin A_1_; GABARAP: GABA type A receptor-associated protein; GABARAPL1: GABA type A receptor associated protein like 1; GFP: enhanced green fluorescent protein; KO: knockout; LDS: LIR docking site; LIR: LC3-interacting region; MAP1LC3/LC3: microtubule associated protein 1 light chain 3; PIK3C3: phosphatidylinositol 3-kinase catalytic subunit type 3; PIK3R4: phosphoinositide-3-kinase regulatory subunit 4; PtdIns3K: phosphatidylinositol 3-kinase; PtdIns3P: phosphatidylinositol-3-phosphate; SQSTM1/p62: sequestosome 1; VPS: Vacuolar protein sorting; ULK: unc-51 like autophagy activating kinase

## Introduction

Autophagy is an evolutionarily conserved intracellular renovation process, responsible for degradation and recycling of dysfunctional or misfolded proteins as well as aged and/or damaged organelles. Macroautophagy (hereafter referred to as autophagy) involves the formation of a double-membrane structure in the cytoplasm, called the phagophore, which expands and closes upon itself to sequester part of the cytoplasm to form an autophagosome. The autophagosome then fuses with a lysosome, forming an autolysosome where the content is degraded and then recycled to the cytosol [1]. Autophagy is induced in response to nutrient limitation or accumulation of damaged proteins and organelles. To date, more than 40 autophagy-related (*Atg*) genes encoding components of the autophagy machinery have been identified by yeast genetics [2]. Most of these are conserved in mammals and regulate the key steps in autophagosome formation, including initiation, nucleation, elongation, lysosome fusion, and degradation. Initiation of autophagy is mediated by the ULK1 (unc-51 like autophagy activating kinase 1) protein kinase complex and the class III phosphatidylinositol 3-kinase complex I (PtdIns3K-C1) [3]. In both yeast and mammals there are at least 2 PtdIns3K complexes responsible for generating phosphatidylinositol 3-phosphate (PtdIns3P)-rich membranes [4,5]. Both complexes consist of a core composed of PIK3C3, PIK3R4 and BECN1 (Vps30/Atg6 in yeast). Complex I, which is involved in autophagy initiation, harbors ATG14 together with the core. Complex II contains UVRAG (UV radiation resistance associated; Vps38 in yeast) instead of ATG14 and has a role in later stages of the autophagy process [6]. PtdIns3P-rich membranes act as platforms for autophagosome nucleation and recruitment of downstream effectors such as ZFYVE1/DFCP1 (double FYVE‐containing protein 1) and WIPIs (WD repeat domain, phosphoinositide interacting) [7–9].

**Figure 1.**
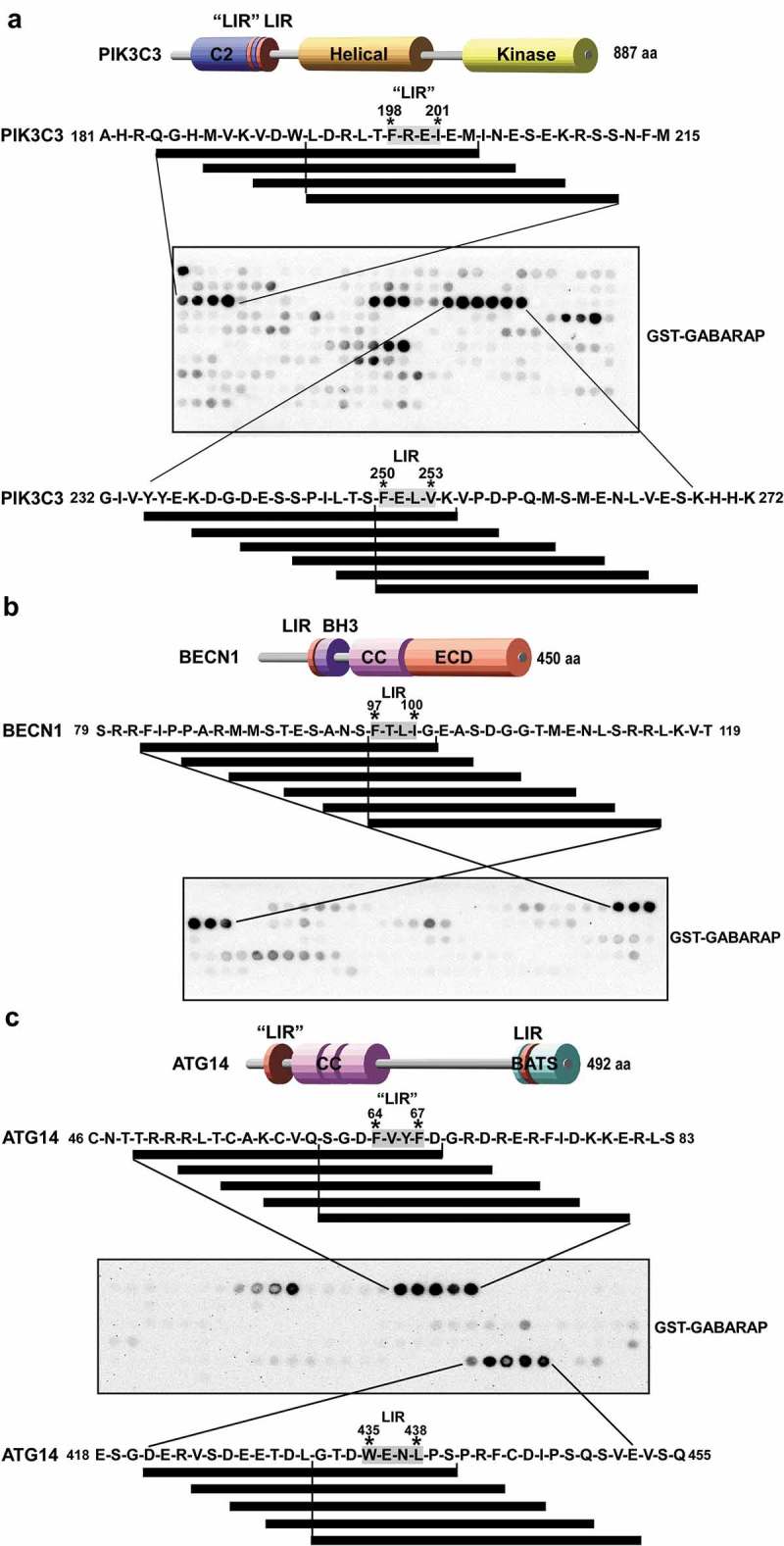
Peptide array analysis indicating LIR motifs in PIK3C3, BECN1 and ATG14. An array of 20-mer peptides covering the full-length of PIK3C3, BECN1 or ATG14, was synthesized on cellulose membrane. Each peptide was shifted 3 amino acids relative to the previous peptide. The array was probed with 1 µg/ml of GST-GABARAP for 2 h and binding was detected with anti-GST antibody. The amino acid sequence for the GABARAP interacting peptides is shown with the interacting peptides depicted as black lines. (**a**) Identification of two putative LIR motifs in PIK3C3 and a schematic representation of the domain organization of human PIK3C3. The locations of LIR-F198 (‘LIR’) and LIR-F250 (LIR) are indicated. (**b**) Identification of a minimal LIR motif in BECN1 and a schematic representation of the domain organization of human BECN1 with the LIR motif indicated. (**c**) Detection of two putative LIR motifs in ATG14. The domain organization of human ATG14 is schematically depicted with the locations of LIR-F64 (‘LIR’) and LIR-W435 (LIR) indicated. BH3: BCL2 homology (BH) domain 3. CC: coil-coil domain. ECD: evolutionarily conserved domain. BATS: Barkor/ATG14 autophagosome targeting sequence domain.

The domain structures and interactions of the individual components organizing the PtdIns3K complexes provide insights into their function on membranes. PIK3R4 is a serine/threonine kinase that contains an N-terminal kinase domain (whose function and possible substrates are uncertain) a C-terminal WD-40 domain as well as several HEAT repeats connecting the kinase and WD-40 domains [10,11]. PIK3C3 consists of an N-terminal C2 domain (implicated in membrane binding), a helical domain and a C-terminal kinase domain [12] (Figure 1(a)). BECN1 contains 3 structural domains (Figure 1(b)): a BH3 domain (BCL2 homology [BH] domain 3) at the N terminus (binds to BCL2 family proteins), a central coiled-coil domain (CCD) responsible for homodimerization and binding to ATG14 and UVRAG, and an evolutionarily conserved domain (ECD also termed the BARA domain for β-α repeated, autophagy-specific) involved in membrane binding) [13]. The ATG14 domain architecture consists of an N-terminal CCD region involved in BECN1 binding, and a C-terminal domain called the BARKOR/ATG14 autophagosome-targeting sequence (BATS) domain (Figure 1(c)) involved in sensing membrane curvature and targeting to the autophagosome [14,15]. A model for the subunit architecture and dynamics of the mammalian ATG14-containing PtdIns3K complex I was obtained using single-particle EM and hydrogen-deuterium exchange studies [16]. A 3-dimensional reconstruction of the complex at 28 Å resolution, displays a V-shaped structure with PIK3R4 positioned throughout the complex acting as a scaffold. The PIK3R4 N terminus is located near the PIK3C3 kinase subunit at the tip of the right arm of the V. The PIK3R4 C-terminal domain, the WD-40 domain, forms a donut shaped region in the left arm of the V while the HEAT repeat domain adopts an arch-shaped form at the junction of the V. Here, the PIK3C3 N-terminal C2 domain as well as BECN1 N terminus and ATG14 N terminus are close to the junction of the V. BECN1 is positioned in the left arm of the complex with its C terminus at the tip of the arm. The close association of the N terminus of BECN1 and N terminus of ATG14 indicates that their coiled-coiled domains are parallel to each other. The dynamics of the complex involves ejection of the kinase domain of PIK3C3 where PIK3R4 is capable of pivoting about the KINHEAT-WD40 junction resulting in an open and closed conformation [16]. A crystal structure of 4.4 Å was obtained for the yeast endosomal PIK3C3 complex (Complex II; Vps34, Vps15, Vps30/Atg6 and Vps38) where the subunits form a Y-shaped complex centered on the PIK3C3 C2 domain [6]. The 2 arms of the Y-shaped complex correspond to the V-shape seen by the EM structure of the mammalian complex described above. The crystal structure shows a parallel heterodimer of Vps30-Vps38 through their coiled coils in the left arm, supporting the predicted parallel arrangement of BECN1 and ATG14 in the mammalian complex. In yeast, Atg38 was identified as a component of the PtdIns3K-C1 complex, interacting with Atg14 [17]. NRBF2 (nuclear receptor binding factor 2), the mammalian homolog of Atg38 interacts with the mammalian PtdIns3K-C1 complex [18–20], resulting in a 10 fold more active complex [21]. NRBF2 binds at the base of the V-shaped PtdIns3K structure, close to the ATG14 and BECN1 N termini, and changes the protein range of motion. NRBF2 drives dimerization of PtdIns3K-C1 converting the active form of the complex to a homodimer of heteropentamers [21].

ATG14 is the autophagy specific component of PtdIns3K-C1 and is responsible for targeting the complex to sites of autophagy initiation [4,22]. The N-terminal cysteine repeats of ATG14 are essential for ER localization [23]. The C-terminal BATS domain is responsible for autophagosome targeting, sensing membrane curvature and binding directly to membranes through an amphipathic alpha helix. The BATS domain binds both PtdIns3P and PtdIns(4,5)P_2_ [15]. In addition to its role in initiation, ATG14 has been implicated in autophagosome-endolysosome fusion. Here, the N-terminal cysteine repeats are essential for the self-association of ATG14 that is crucial for interaction with STX17 and enhancing SNARE (soluble N-ethylmaleimide-sensitive factor attachment protein receptors) complex-mediated membrane fusion. Furthermore, the BATS domain is also required for ATG14-mediated membrane tethering [14]. A recent report shows that binding of the BATS domain to PtdIns(4,5)P_2_ regulates the interaction of ATG14 with PIK3C3 and BECN1, influencing complex assembly and autophagy initiation [24]. Phosphorylation of S29 in the N terminus of ATG14 by ULK1/2 is of vital importance for activation of the lipid kinase activity of the PtdIns3K-C1 complex both upon amino acid starvation, glutamine deprivation and during hypoxia [25–27].

During autophagosome formation, a small ubiquitin-like (Ubl) protein termed Atg8 in yeast is conjugated to phosphatidylethanolamine (PE) on the membranes of the phagophore in a process termed Atg8 lipidation. In mammals, the Atg8 homologs constitute 2 subfamilies based on amino-acid-sequence homology: MAP1LC3/LC3 (microtubule associated protein 1 light chain 3) family with LC3A (2 N-terminal splice variants), LC3B, LC3B2 and LC3C and the GABARAP (γ-aminobutyric acid receptor-associated protein) family with GABARAP, GABARAPL1 and GABARAPL2 [28]. LC3B is a well-established autophagosome marker. The Atg8-family proteins promote the expansion of the phagophore membrane as well as its closure and fusion with the lysosome [2,28,29]. Conjugated Atg8 proteins function as scaffolds, to recruit various proteins to the phagophore membrane [30]. For many of the recruited proteins, the interaction with Atg8 homologs is mediated by an LC3-interacting region (LIR) motif, a short linear motif of up to 13 amino acids. The core LIR sequence complies with the generic formula φ-X-X-η—where φ is an aromatic amino acid (W/F/Y), η is hydrophobic (L/I/V), and X can be any amino acid [30,31]. The LIR motif was first identified in the cargo receptor SQSTM1/p62 (sequestosome 1) [32–34]. The Atg8-family proteins have a C-terminal, ‘core’ Ubl domain with the conserved ‘ubiquitin fold’ and an additional N-terminal arm with two α-helices that are closed onto the core Ubl domain. In the interface of the N-terminal arm and the Ubl domain resides the LIR docking site. Two hydrophobic pockets, HP1 and HP2, in the Ubl domain of Atg8 ensure binding of the side chains of the φ and η residues of the core LIR [35]. A growing list of proteins in yeast and mammals, other than cargo receptors, contain functional LIR motifs [30,31,36]. These include proteins of the core autophagy machinery such as yeast Atg1 [37,38], components of the mammalian ULK1/2 complex [39], yeast Atg3 [40], yeast Atg4 [41] and mammalian ATG4B [42,43].

In this study we set out to identify LIR motifs in the PtdIns3K complex I. We revealed functional LIR motifs in PIK3C3, BECN1 and ATG14, applying peptide arrays and GST-affinity-isolation assays. For all complex I proteins, the LIRs displayed a preference for interaction with GABARAP and GABARAPL1. We obtained high-resolution crystal structures of the LIR peptide complexes for PIK3C3 and BECN1 with GABARAP and for ATG14 with GABARAPL1, respectively. All the LIR motifs identified are canonical LIR motifs binding to the two hydrophobic pockets of GABARAP and GABARAPL1. The crystal structures correlated very well with the amino acid specificity preferences of the LIR motifs demonstrated by 2D peptide arrays and suggest how specificity towards binding to the GABARAP proteins is achieved. ATG14 is unique to the autophagy-specific PtdIns3K complex I. We therefore focused functional studies on ATG14. Mutation of the LIR motif of ATG14 still allowed formation of the PtdIns3K-C1 complex. However, colocalization with LC3B was blocked and mitophagy impaired. Furthermore, ULK-mediated phosphorylation of S29 in ATG14, shown to be very important for activation of the PtdIns3K-C1 complex, was strongly reduced. Hence, the GABARAP-preferring LIR motifs in PIK3C3, BECN1, and particularly ATG14, help scaffolding the PtdIns3K-C1 on membranes to facilitate efficient autophagosome formation.

## Results

### PIK3C3, BECN1 and ATG14 contain putative LIR motifs

In order to reveal putative LIR motifs in components of PtdIns3K–C1 we conducted peptide array analysis for each protein (PIK3C3, BECN1 and ATG14), except for PIK3R4, which did not show significant binding to Atg8-family proteins in pull down assays (Fig. S1A). Briefly, an array of 20-mer peptides that cover the full-length protein, with a spacing of 3 amino acids, was synthesized on a cellulose membrane and probed with recombinant GST or GST-GABARAP. Binding was then detected with anti-GST antibodies [39]. The peptide array for PIK3C3 indicated two potential LIR motifs; a minimal motif of 11 residues with the sequence LDRLTFREIEM (LIR-F198) and a second minimal motif of 5 residues with the sequence FELVK (LIR-F250). Both of these motifs are located near the C-terminal end of the C2 domain of PIK3C3 (Figure 1(a); core amino acids F198-I201 denoted ‘LIR’ and F250-V253 denoted LIR, respectively). In a similar manner we mapped the minimal LIR motif of BECN1 to the 5-amino acid sequence of FTLIG. This putative LIR motif is adjacent to the BH3 domain of BECN1 (Figure 1(b); amino acids F97-I100). The peptide walk for ATG14 revealed two potential LIR motifs: A minimal motif of 7 residues with the sequence GDFVYFD (LIR-F64 denoted ‘LIR’, core amino acids F64-F67) and a 7-amino acid sequence of TDWENLP (LIR-W435 denoted LIR, core amino acids W435-L438) (Figure 1(c)). The LIR-F64 motif is within the first 70 amino acids of ATG14 while LIR-W435 is located within the last 80 amino acids comprising the BATS domain.

### PIK3C3, BECN1 and ATG14 contain functional LIR motifs and interact with the Atg8-family proteins with a preference for GABARAP and GABARAPL1

**Figure 2.**
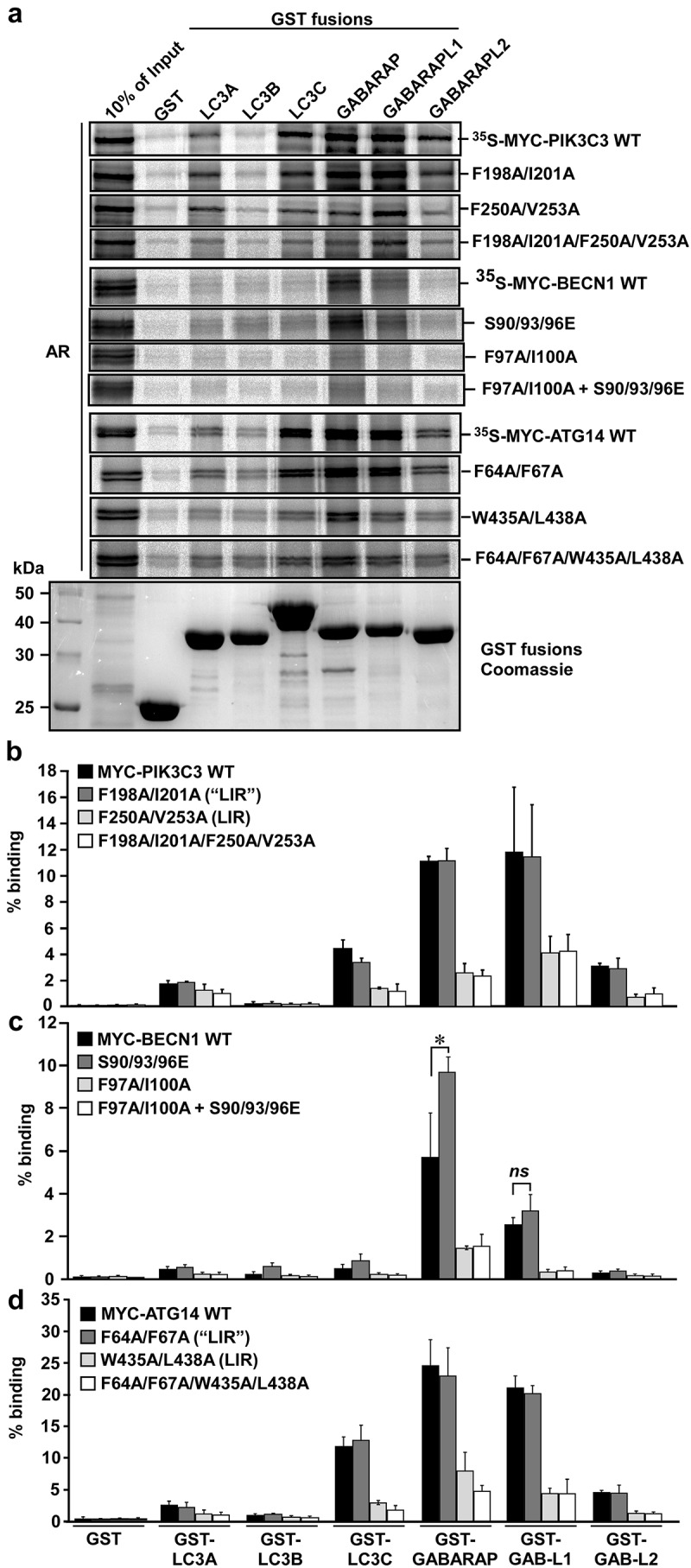
Verification of functional LIR motifs in PIK3C3, BECN1 and ATG14. The putative core LIR motifs, identified using peptide array analysis, were mutated by site-directed mutagenesis in the context of the full-length protein. Point mutations of both the aromatic residue and the conserved hydrophobic position were introduced in order to replace these residues with alanine. The impact of mutating the LIR motifs was then assessed in GST-pulldown analyzes with recombinant GST-Atg8 homologs and *in vitro*-translated ^35^S-labelled Myc-tagged PIK3C3, BECN1 and ATG14. Phosphomimetic mutations of the previously identified important serines (S90E, S93E and S96E) of BECN1 were made and tested for functionality with the identified LIR motif FTLI or a LIR mutant. (**a**) Autoradiographs (AR, top panels) and Coomassie Brilliant Blue-stained immobilized GST or GST-fusion proteins (bottom panel) are shown. (**b-d**) Quantifications of the binding of wild-type and mutant constructs to the GST-Atg8 proteins is presented as percentage binding relative to the 10% input. There is a significant increase in binding of the phosphomimetic mutant of BECN1 in (c). Data information: Means ± SD of 3 independent experiments. Significant *P* value is indicated as * = *P* value is 0.017 (Student´s two-tailed, unpaired *t*-test). ns, not significant.

The putative core LIR motifs of PIK3C3, BECN1 and ATG14 identified using peptide array analysis were subjected to site-directed mutagenesis in the context of full-length proteins. The impact of mutating the LIR motifs was then assessed in GST-affinity-isolation assays. Here, the different Atg8 homologs were expressed as GST fusions in *E. coli* and then tested for interaction with *in vitro* translated PIK3C3, BECN1 and ATG14, respectively. All the members of PtdIns3K-C1, except PIK3R4, were able to interact with the Atg8 homologs (Figure 2). PIK3C3 and ATG14 showed significant interaction with LC3C and GABARAPL2, but the strongest interaction was seen with GABARAP and GABARAPL1. BECN1 only bound with significant affinity to GABARAP and GABARAPL1. Taken together, PIK3C3, BECN1 and ATG14 all interacted with the Atg8 homologs and appear to have a preference for binding to the GABARAP and GABARAPL1. For PIK3C3, point mutations of both the aromatic residue and the conserved hydrophobic position (F250A, V253A) of the core LIR-F250 motif (FELV, amino acids 250–253) appeared to significantly reduce binding to the Atg8 homologs. In contrast, mutating the core LIR-F198 motif (FREI, amino acids 198–201) in a similar manner (F198A, V201A) did not affect binding of PIK3C3 (Figure 2). This indicates that LIR-F250 of PIK3C3 is the functional LIR motif. The putative core motif of BECN1 (FTLI amino acids F97-I100) was also mutated (F97A, I100A) and tested for interaction with the recombinant GST-Atg8 homologs. The mutations significantly reduced binding and confirmed a functional LIR motif in BECN1 (Figure 2). Three serine residues S90, S93 and S96, just preceding the LIR motif of BECN1, have previously been identified as sites of phosphorylation [44,45]. In light of these data we wanted to investigate whether binding of BECN1 to the Atg8 homologs was influenced by phosphorylation of these 3 serine residues. We performed site-directed mutagenesis of all the 3 sites (S90E, S93E, S96E) in BECN1 and the BECN1 LIR mutant, resulting in phosphomimetic mutants. We then tested these mutants for binding to Atg8 homologs in GST-affinity-isolation assays. These phosphomimetic mutations increased binding of BECN1 to the Atg8 homologs but showed no effect in the LIR mutant (Figure 2). The GST-pulldown assays of the mutated core LIR motifs of ATG14, LIR-F64 (FVYF mutated to F64A/F67A) and LIR-W435 (WENL mutated to W435A, L438A) identified the LIR-W435 motif in the BATS domain as the major LIR motif of ATG14 (Figure 2).

We then used biolayer interferometry to determine the binding affinities of biotin-labeled 16-mer LIR peptides binding to recombinant LC3A, -B, -C, GABARAP and GABARAPL1 (Table 1). The Kd measurements for the LIR peptides were completely consistent with the GST-affinity-isolation data with GABARAP and GABARAPL1 as the strongest interactors with the LIRs of ATG14, BECN1 and PIK3C3. The ATG14 LIR had the strongest interaction with Kds of 2.85 μM and 1.9 μM for binding to GABARAP and GABARAPL1, respectively, followed by BECN1 (Kds of 10.5 μM and 7.95 μM) with PIK3C3 as the weakest binder with Kds of 49.5 μM and 65 μM (Table 1). The binding to LC3B was very weak for all 3 LIRs while ATG14 LIR bound well to LC3C (Kd 5.85 μM). The affinity of the ATG14 LIR peptide was similar to the SQSTM1 LIR interacting with LC3B (1.5 μM) as determined by isothermal calorimetry [46]. The higher affinity observed for the ATG14 LIR motif compared to BECN1 and PIK3C3 is most likely due to the presence of a tryptophan in position 0. Indeed, it has been shown previously that a LIR motif with a tryptophan in position 0, occupying HP1, binds more strongly to Atg8-family proteins than the same LIR with a phenylalanine or tyrosine at this position [46]. Phosphomimetic mutations of BECN1 at S93 and S96 increased the binding affinity 3-fold for GABARAP and GABARAPL1, 8-fold for LC3C and 5-fold for LC3A. The most striking effect was seen for the phosphomimetic mutations at S244 and S249 for PIK3C3, both sites known to be phosphorylated (https://www.phosphosite.org), the latter by ULK1 [47], where binding increased 17-, 19- and 15-fold to GABARAP, GABARAPL1 and LC3C, respectively (Table 1). As shown in peptide array binding assays, the increased binding and broadened specificity of the S249E mutant of PIK3C3 extended to include LC3B. Likewise, S93E and S96E of BECN1 also displayed stronger binding in these assays (Fig. S1B).

**Table 1.** Affinities (K_D_ values in µM) of LIR peptides binding to ATG8 family members*.

**Figure 3.**
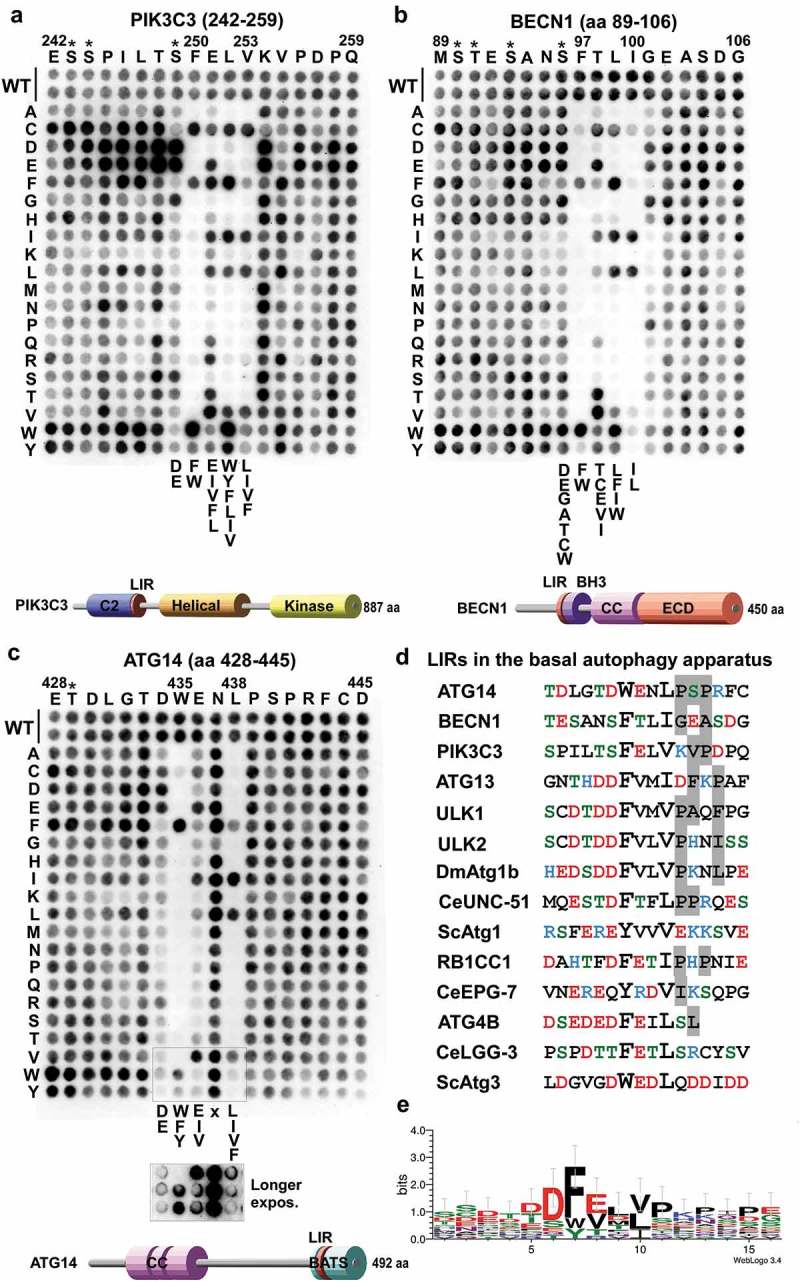
Substitution analyzes of the binding of GABARAPL1 to the LIR motifs of PIK3C3, BECN1 and ATG14 using 2D peptide arrays. Two-dimensional peptide arrays scans analyzing the effect of single amino acid substitutions at all positions of the indicated 18-mer peptides containing the functional LIR motifs from (**a**) PIK3C3 (amino acids 242–259); (**b**) BECN1 (amino acids 89–106) and (**c**) ATG14 (amino acids 428–445). Each position of the 18-mer peptides was replaced with all 20 amino acids. The array was probed with 1 µg/ml of GST-GABARAPL1 for 2 h and binding to GST-GABARAPL1 was detected with anti-GST antibody. Asterisks indicate the location of known phosphosites. (**d**) Alignments of the functional LIR motifs identified in components of the PtdIns3K complex I in this work with those previously identified in the human ULK1/2 complex, *Drosophila* Atg1b/DmATG1B, *C. elegans* UNC-51 (CeUNC-51), *S. cerevisiae* Atg1 (ScAtg1), human ATG4B, *C. elegans* Lgg-3 (CeLGG-3) and *S. cerevisiae* Atg3 (ScAtg3). Residues at positions C-terminal to the core LIR motif that are known to interact via hydrophobic interactions with HP2 (from this study) or may do so are boxed in gray. (**e**) A sequence logo illustrates the distribution of amino acids at each position of the aligned sequences.

*The sequences of the peptides used are shown in Figure 3(d) with the BECN1 S2E peptide containing S to E substitutions of the **S**AN**S** sequence (corresponding to S93 and S96), and PIK3C3 S2E containing S to E substitutions of the **S**PILT**S** sequence (corresponding to S244 and S249), immediately N-terminal to the core LIR motifs.

The identified functional LIR motifs of PIK3C3, BECN1 and ATG14 were tested for specificity in 2-dimensional (2D) peptide array scans using GST-GABARAPL1. Here, the importance of each position of the LIR motifs of PIK3C3, BECN1 and ATG14 was revealed, using peptide arrays with a series of 18-mer peptides with each core LIR motif in the middle, each peptide containing single point mutations. All 18 positions were mutated to all alternative amino acids, and the effects of the different point mutations on binding to GST-GABARAPL1 were then tested (Figure 3). The 2D peptide array of the PIK3C3 LIR showed that it is a canonical F-type (F at the aromatic HP1 position) LIR with some stringent requirements. At the aromatic HP1 position only W was tolerated as a substitution for F. At the hydrophobic HP2 position I was better tolerated than L. Interestingly, at position +1 of the core LIR E could not be substituted by D, but by the hydrophobic residues I,V, L and F. At position +2 only the 3 large hydrophobic residues L, I, V, or the 3 aromatic residues were accepted. This position is usually the promiscuous position where most substitutions, except for H, R, K, P or G are allowed. A striking preference for substitutions to acidic D or E residues is seen at positions −1 to −7 N-terminal to the core LIR. Known phosphorylation sites (indicated by asterisks) are present at 3 of these positions (S243, S244 and S249). The S249 site is reported to be phosphorylated by ULK1 [47](Figure 3(a)).

For the BECN1 LIR, the 2D peptide array showed that it is a canonical LIR, but it was much more sensitive to substitutions in all four core LIR positions than usually seen. The F at HP1 could be effectively substituted by W, but not by Y. At the hydrophobic +3 position only a change to L is allowed. Both the +1 and +2 positions were relatively sensitive to most substitutions with C, E, I and V being well tolerated at the +1 position and F, I and W well tolerated at the +2 position. The G101 at position +4 was only well substituted by D, E and P. The S96 (a known phosphosite) was well substituted by A, C, D, E, G, T and W (Figure 3(b)).

For the ATG14 LIR, the 2D peptide array showed that it is a canonical LIR with aromatic substitutions accepted at the 0 position, although a longer exposure was necessary to see this clearly because of weaker signals at the bottom two rows of the array. At the hydrophobic +3 position also F was allowed, but not M. There was a requirement for D or E at the −1 position, and at the +1 position I or V could substitute for E, while D could not (Figure 3(c)).

### High-resolution crystal structures of the functional LIR motifs in PtdIns3K-C1

**Figure 4.**
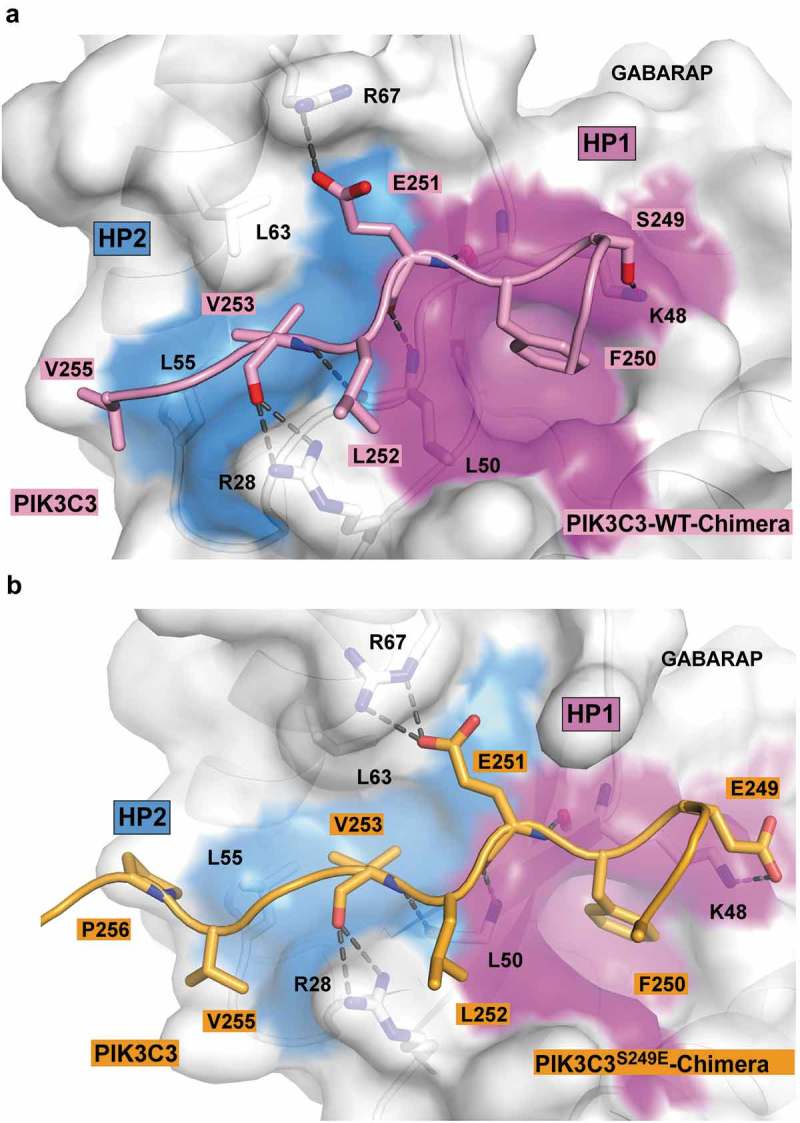
Structure of wild-type and PIK3C3^S249E^ LIR motif bound to GABARAP. (**a**) Close-up of the chimera structure for the wild-type PIK3C3 LIR motif bound to GABARAP. The LIR of PIK3C3 (amino acids 244–258) is displayed in pink cartoon with the interacting residues shown as sticks. GABARAP is displayed in white cartoon and transparent surface with the hydrophobic pocket 1 and 2 colored in pink and blue surfaces, respectively. (**b**) Close-up of the chimera structure for PIK3C3^S249E^ LIR motif bound to GABARAP. The LIR of PIK3C3 (amino acids 244–258) containing the phopshomimetic mutation S249E is displayed in orange cartoon with the interacting residues shown as sticks. GABARAP is displayed in white cartoon and transparent surface with the hydrophobic pocket 1 and 2 colored in pink and blue surfaces, respectively.

**Figure 5.**
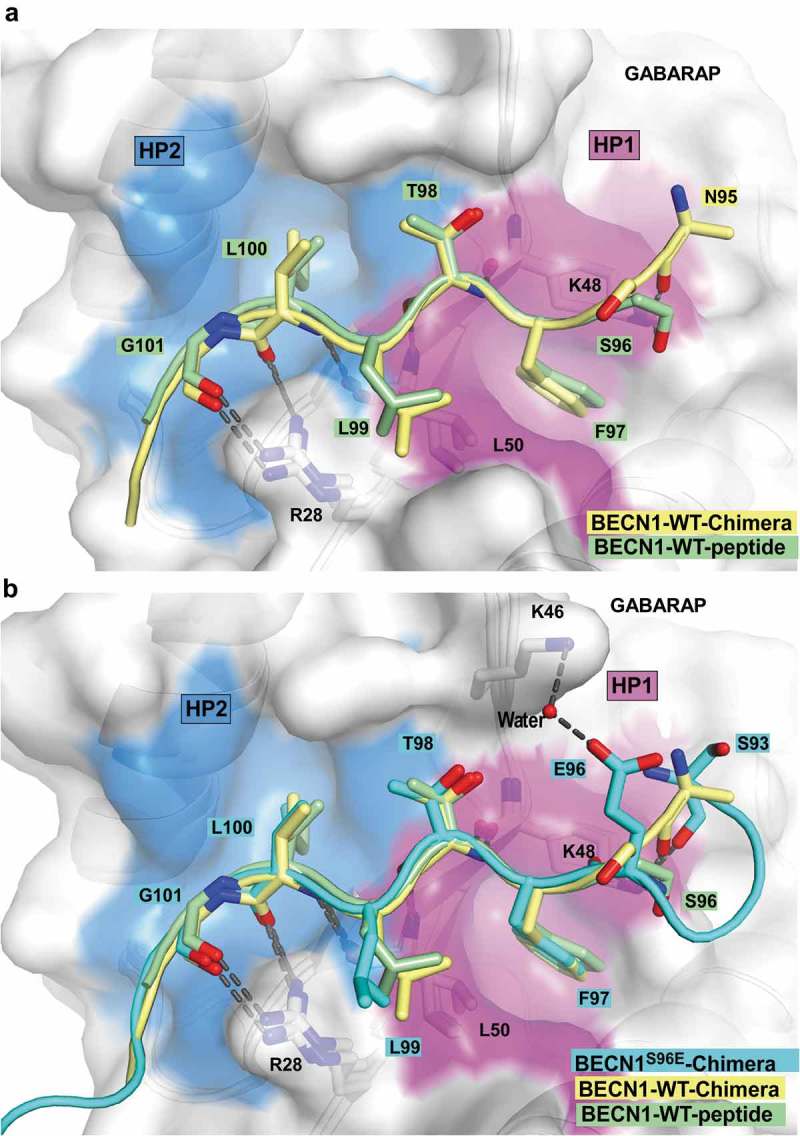
Structure of the BECN1 LIR motif bound to GABARAP and GABARAPL1. (**a**) Superposition of the chimera structure of wild type BECN1 LIR motif (yellow) bound to GABARAP and the wild type BECN1 LIR peptide (green) bound to GABARAPL1. Both LIR motifs are displayed in cartoon with the interacting residues shown as sticks. GABARAP and GABARAPL1 are displayed in white cartoon and GABARAP is displayed as well in transparent surface with the hydrophobic pocket 1 and 2 colored in pink and blue surfaces, respectively. (**b**) Same as (A) with the chimera structure of BECN1^S96E^ LIR motif (cyan) bound to GABARAP superposed.

**Figure 6.**
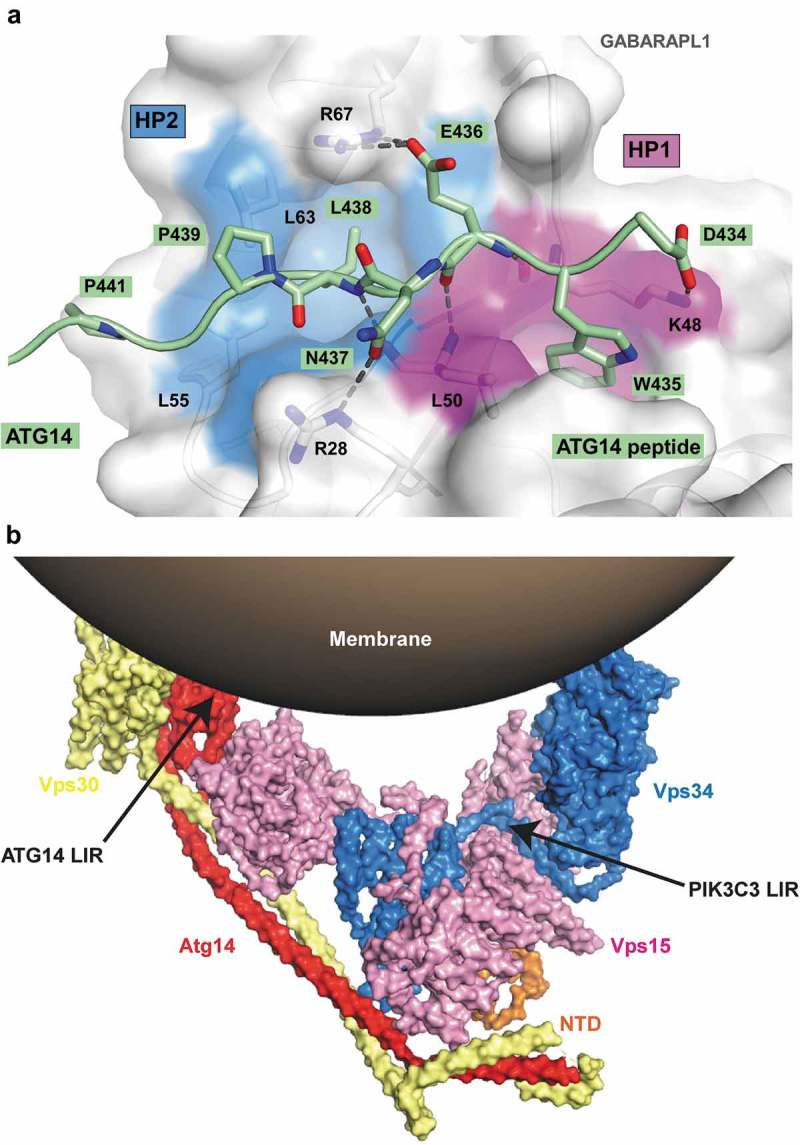
Structure of ATG14 LIR-W435 motif bound to GABARAPL1. (**a**) Close-up of the peptide structure for wild-type ATG14 LIR motif bound to GABARAPL1. The LIR of ATG14 (amino acids 429–443) is displayed in green cartoon with the interacting residues shown as sticks. GABARAPL1 is displayed in white cartoon and transparent surface with the hydrophobic pocket 1 and 2 colored in pink and blue surfaces, respectively. (**b**) Model of yeast complex I containing the PIK3C3 homolog Vps34, PIK3R4 homolog Vps15, BECN1 homolog Vps30 and Atg14 (PDB ID: 5DFZ) on a curved membrane [6]. The membrane-proximal localization position of ATG14 and PIK3C3 LIR motifs is indicated on the figure. The structure of the part of BECN1 containing the LIR motif is not solved and therefore impossible to localize on the model.

To provide a molecular basis for the binding of these LIR motifs to Atg8-family proteins, we determined the crystal structure of the WT LIR motifs of PIK3C3 (Figure 4(a)), BECN1 (Figure 5(a)), and ATG14 (Figure 6(a)), as well as the phosphomimetic S249E for PIK3C3 (Figure 4(b)), and S96E for BECN1 LIR motifs (Figure 5(b)). We used two strategies to obtain the complex structures. We first tried to co-crystallize GABARAPL1 with a LIR peptide. If that did not work we made a chimeric protein consisting of GABARAP N-terminally fused to the LIR sequence with a Gly-Ser linker. Co-crystals of GABARAPL1 with WT LIR peptides diffracted to 1.14 Å for BECN1[93–102], and 1.4 Å for ATG14[429–443]. Crystals of GABARAP chimeras diffracted to 1.26 Å for WT PIK3C3[244–258], 2.25 Å for S249E PIK3C3[244–258], 1.51 Å for WT BECN1[93–105] and 1.61 Å for S96E BECN1[93–105] (Table 2 and Fig. S2-4).

**Table 2.** Data collection and refinement statistics.

	PIK3C3 WT (Chimera)	PIK3C3^S249E^ (Chimera)	BECN1 WT (peptide)	BECN1 WT (Chimera)	BECN1^S96E^ (Chimera)	ATG14 (peptide)
	6HOG	6HOH	6HOI	6HOJ	6HOK	6HOL
**Data Collection**						
Resolution range	28.66–1.26 (1.30–1.26)	53.44–2.25 (2.33–2.25)	35.16–1.14 (1.18–1.14)	53.7–1.51 (1.56–1.51)	49.72–1.61 (1.66–1.61)	41.48–1.40 (1.45–1.40)
Space group	H3	P 2_1_ 3	P 2_1_ 2_1_ 2_1_	P 2_1_ 2_1_ 2	I 4_1_ 3 2	P 1
Unit cell	92.1 92.1 41.2 90 90 120	119.5 119.5 119.5 90 90 90	31.9 54.0 138.9 90 90 90	76.6 150.4 35.5 90 90 90	140.6 140.6 140.6 90 90 90	34.4 41.6 46.2 86.0 76.2 86.7
Total reflections	118 617 (10 172)	158 208 (15 830)	357 779 (24 004)	340 733 (24 654)	209 878 (21 149)	110 346 (10 921)
Unique reflections	34 959 (3 478)	27 219 (2 654)	88 324 (8 544)	65 391 (6 408)	30 838 (2 999)	45 978 (4 496)
Multiplicity	3.4 (2.9)	5.8 (5.9)	4.1 (2.8)	5.2 (3.8)	6.8 (7.0)	2.4 (2.4)
Completeness (%)	99.17 (98.19)	99.49 (99.21)	99.34 (97.51)	99.50 (98.89)	99.01 (96.84)	93.30 (90.33)
Mean I/sigma(I)	19.2 (1.5)	10.4 (1.6)	18.2 (1.7)	13.2 (1.2)	17.1 (1.5)	6.2 (1.3)
Wilson B-factor	18,4	39,0	14,0	21,2	28,8	15,1
R-merge	0.02 (0.73)	0.08 (0.98)	0.03 (0.64)	0.06 (1.02)	0.04 (1.16)	0.07 (0.71)
R-meas	0.03 (0.89)	0.09 (1.07)	0.03 (0.78)	0.07 (1.18)	0.04 (1.26)	0.10 (0.90)
R-pim	0.01 (0.49)	0.03 (0.44)	0.01 (0.44)	0.03 (0.57)	0.01 (0.47)	0.06 (0.55)
CC1/2	0.99 (0.53)	0.99 (0.65)	0.99 (0.58)	0.99 (0.58)	0.99 (0.61)	0.99 (0.46)
**Refinement**						
Reflections used in refinement	34 958 (3 478)	27 048 (2 637)	88 324 (8 545)	65 386 (6 405)	30 604 (2 939)	45 714 (4 422)
Reflections used for R-free	1 581 (131)	1 272 (119)	4 387 (427)	3 254 (309)	1 998 (195)	2 214 (221)
R-work	0.15 (0.28)	0.20 (0.34)	0.14 (0.24)	0.16 (0.26)	0.16 (0.36)	0.16 (0.22)
R-free	0.18 (0.33)	0.24 (0.38)	0.16 (0.26)	0.21 (0.29)	0.19 (0.41)	0.20 (0.29)
Number of non-hydrogen atoms	1 166	4 362	2 357	3 474	1 157	2 393
macromolecules	1 054	4 192	2 158	3 109	1 059	2 188
ligands	30	10	16	9	8	21
solvent	82	160	183	356	90	184
Protein residues	123	506	251	371	127	252
RMS(bonds)	0.005	0.002	0.01	0.013	0.01	0.015
RMS(angles)	0.79	0.44	1.18	1.22	1.03	1.35
Ramachandran favored (%)	99.2	98.6	99.1	98.4	98.4	97.9
Ramachandran allowed (%)	0.8	1.4	0.8	1.3	1.6	2.1
Ramachandran outliers (%)	0	0	0	0	0	0
Average B-factor (all)	28.8	50.7	20.3	27.7	42.2	23.4
macromolecules	27	50.8	19.3	26.4	41.1	22.5
ligands	52	78.5	35.5	42	61.5	44
solvent	41.1	47.8	29.8	38.1	53.7	31.6

PIK3C3, BECN1 and ATG14 WT LIR motifs bound to GABARAPL1 in and extended conformation with the two core hydrophobic residues in position 0 and +3 deeply bound to the hydrophobic pockets HP1 and HP2. They all display 3 conserved hydrogen bonds in-between the main chain of the residues in position +1 and +3 of the LIR motif and GABARAPL1 residues K48 and L50. These interactions are previously observed for canonical LIR interactions [35]. However, each of these 3 LIR motifs displayed some additional specific interactions described below.

The additional contacts observed in the WT PIK3C3-GABARAP complex were: 1) A salt bridge between E251 (PIK3C3) and R67 (GABARAP); 2) hydrophobic contacts between L252 (PIK3C3)-V255 (PIK3C3) with the edge of the hydrophobic pocket 1 and 2, respectively; 3) Two hydrogen bonds between the guanidinium group of R28 (GABARAP) and the carbonyl of V253 (PIK3C3) main chain, as well as a third hydrogen bond between the side chain of S249 (PIK3C3) and K48 (GABARAP) (Figure 4(a,b)). The structure provides an explanation for the requirement of a glutamate (E) in position +1 observed in the peptide array (Figure 3(a)).

The specific interactions observed in WT BECN1 structures were: 1) Hydrophobic contact between L99 (BECN1) in position +2 and the edge of HP2; 2) two hydrogen bonds between the guanidine of R28 (GABARAPL1) and the carbonyls of G101 (BECN1)-L100 (BECN1), as well as a third one between the carbonyl of N95 (BECN1) (or the side chain of S96 for the peptide structure) and the side chain of K48 (GABARAPL1) (Figure 5(a,b)).

The ATG14 LIR-GABARAPL1 structure displayed the following additional interactions: 1) 2 salt bridges between the acidic residues D434 (ATG14), E436 (ATG14) and the basic residues K48 (GABARAPL1), R67 (GABARAPL1), respectively; 2) a hydrogen bond between the side chain of N437 (ATG14) and the guanidinium group of R28 (GABARAPL1); 3) some hydrophobic interactions between P439 (ATG14)-P441 (ATG14) and L55 (GABARAPL1)-L63 (GABARAPL1) from HP2 (Figure 6(a)). The ATG14 peptide array confirmed that the two salt bridges formed by D434 (ATG14) and E436 (ATG14) are both critical for LIR binding (Figure 3(c)).

The determination of the crystal structures of GABARAP chimeras containing phosphomimetic LIR mutations S249E in PIK3C3[244–258] and S96E in BECN1[93–105] provided us with valuable information to explain the increased binding observed upon phosphorylation. The S96E BECN1 LIR bound GABARAP similarly to the WT LIR (Figure 5(b)). However, E96 formed an additional water bridged hydrogen bond with K46 (GABARAP), which could explain the increased affinity of the phosphorylated LIR. The PIK3C3^S249E^ LIR bound GABARAP similarly to the WT PIK3C3 LIR in its C-terminal moiety (from residue F250 [PIK3C3] to P256 [PIK3C3]); however, we observe some variation in its N-terminal moiety. E249 (PIK3C3) formed a salt bridge with K48 (GABARAP) (Figure 4(b)).

### Variation in hydrophobic pocket 2 (HP2) may explain the specificity of PIK3C3, BECN1 and ATG14 LIRs for the GABARAP family

The 3 LIR motifs of PtdIns3K-C1 are binding preferentially to the GABARAP family and more weakly to LC3C. In order to understand why they are not binding to LC3A and LC3B we superimposed the LC3B structure (PDB ID:3VTU) onto each of the structures we solved in this study (Fig. S5). In each of those 3 structures we observed some hydrophobic interactions between the residues C-terminal to the core LIR motif in position +4 to +6 (V255 [PIK3C3]-P256 [PIK3C3]; G101 [BECN1]-A103 [BECN1]; P439 [ATG14]-P441 [ATG14]) and HP2 (Figure 3(d)). In LC3A and LC3B, the HP2 showed some variations with F60 (GABARAP) and L55 (GABARAP) forming the bottom of the hydrophobic pocket and interacted with the highly conserved hydrophobic residue in position +3 of the LIRs as well as with the hydrophpobic residues in position +4 to +6. The F60 and L55 residues of GABARAP are replaced by shorter leucine and valine residues in LC3B and LC3A (L63 [LC3A/B] and V58 [LC3A/B]). This may reduce the strength of the interaction between the LIR motifs and the HP2 (Fig. S5). We observed as well that the H57 [LC3B] (equivalent to D54 [GABARAP]) clashed with the residues in position +4 to +6 of each of the LIR motifs. Interestingly, D54 (GABARAP)-L55 (GABARAP) and F60 (GABARAP) are conserved in GABARAPL1, GABARAPL2 and LC3C which bind to PIK3C3, BECN1 and ATG14 LIR motifs. We are therefore proposing that these 3 residues are responsible for the specificity of the PtdIns3K-C1 LIR motifs to GABARAPs and LC3C. A similar observation has been made previously for the role of H57 in LC3A/LC3B and D54 in GABARAPs/LC3C for the WDFY/ALFY (WD repeat and FYVE domain containing 3)[3342–3354] LIR motif which binds specifically to GABARAPs/LC3C and FYCO1 (FYVE and coiled-coil domain containing 1)[1276–1288], which binds specifically to LC3A/LC3B [48,49].

### The ATG14 LIR interaction facilitates binding of BECN1 and PIK3C3 to GABARAP and GABARAPL1

**Figure 7.**
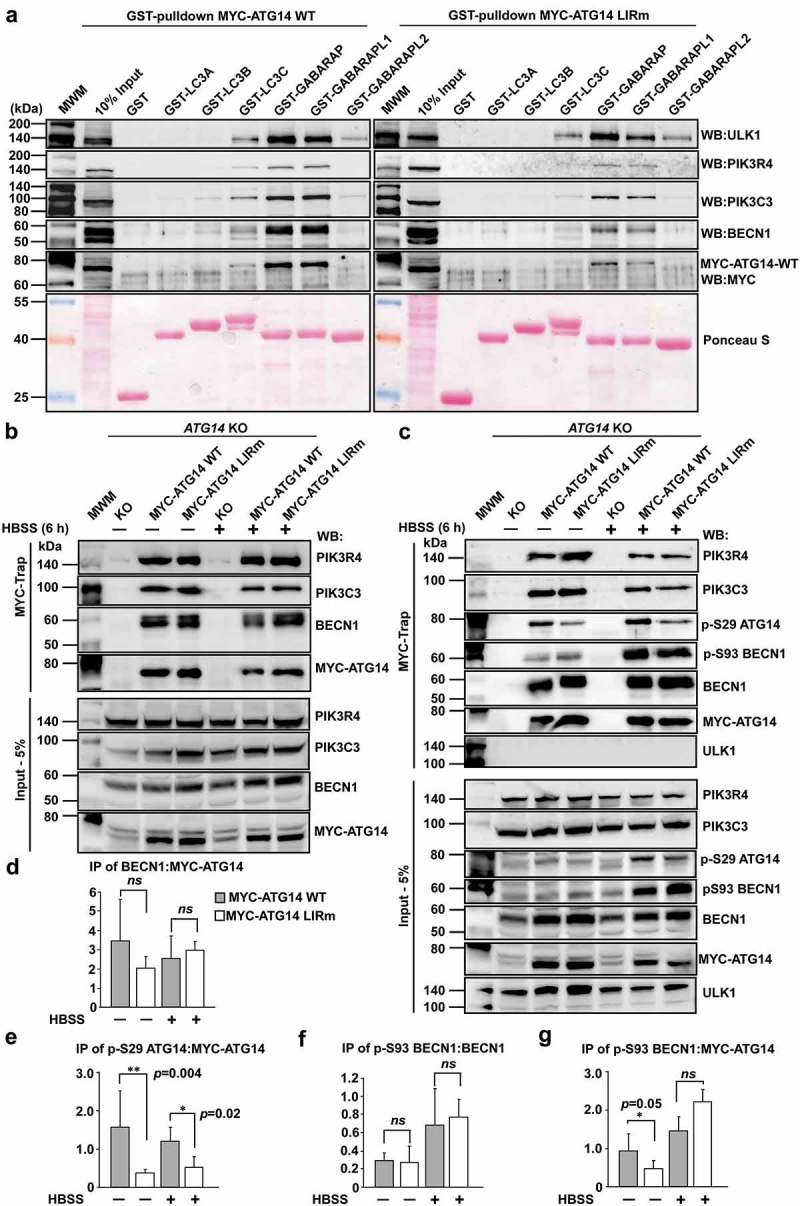
The ATG14 LIR motif is important for binding of the PtdIns3K-C1 complex to GABARAP and GABARAPL1, and for activation of the complex by ULK1-mediated Ser29 phosphorylation of ATG14. (**a**) GST affinity isolation using bead-bound GST-Atg8-family fusion proteins and lysates from HCT116 cells knocked out for *ATG14* and reconstituted with WT or LIR mutated MYC-ATG14. Bead bound proteins were detected by western blots using the indicated antibodies. (**b**) MYC-Trap experiments performed with the indicated cell lines. Bound proteins were detected by western blots using the denoted antibodies. (**c**) MYC-Trap experiments performed with the indicated cell lines, incubated in starvation medium (HBSS) for 6 h or not. Bound proteins were detected by western blots using the indicated antibodies. (**d-**g) Relative quantifications of the data in (c), showing the relative amount of BECN1, p-S93 BECN1 and p-S29 MYC-ATG14 in the MYC precipitates. Data information: Means ± SD of 3 independent experiments. Significant *P* values are indicated (Student´s two-tailed, unpaired *t*-test). ns, not significant.

The LIR motifs of ATG14 and PIK3C3 are in regions important for membrane binding of the complex, as displayed in recently solved crystal structures of the entire complex [6] (Figure 6(b)). ATG14 is responsible for targeting the complex to sites of autophagy initiation [4,22]. Given the multiple roles of the PtdIns3K-C1s core components PIK3C3, PIK3R4 and BECN1, in several cellular processes, we focused on ATG14 as the sole autophagy specific component of the complex to directly address a functional role for the LIR motifs. To this end we stably reconstituted human HCT116 cells that were knocked out (KO) for *ATG14* [44], with either MYC-ATG14 WT or MYC-ATG14 LIR mutant constructs expressed at the endogenous level (Fig. S6A). Total cell lysates from the reconstituted cell lines were used in GST affinity-isolation assays to assess binding of MYC-ATG14 as well as endogenous BECN1, PIK3C3 and PIK3R4 and ULK1. Completely consistent with the data obtained in *in vitro* GST affinity-isolation assays (Figure 2), MYC-ATG14 as well as BECN1, PIK3C3 and ULK1 all showed preferential binding to GABARAP and GABARAPL1 while binding was significantly reduced for the ATG14 LIR mutant (Figure 7(a)). Interestingly, binding of BECN1 and PIK3C3 to GABARAP and GABARAPL1 was diminished in the ATG14 LIR mutant cell line (Figure 7(a)) while binding of ULK1 was not affected. This indicates that the binding of the other PtdIns3K complex I members BECN1 and PIK3C3 to GABARAP and GABARAPL1 depends on the LIR interaction mediated by ATG14.

### The LIR motif of ATG14 is important for formation of an active PtdIns3K-C1 complex

To further investigate effects of the ATG14 LIR mutation on PtdIns3K-C1 complex formation, we exploited MYC-Trap for immunoprecipitation (IP) of MYC-ATG14 or MYC-ATG14 LIR mutant from the reconstituted *ATG14* KO cells (Figure 7(b)). The ATG14 LIR mutation does not affect C1 complex formation since similar amounts of BECN1, PIK3C3 and PIK3R4 were present in the IPs of ATG14 WT and LIR mutant (Figure 7(b,d)). Phosphorylation of S29 of ATG14 by ULK1/2 is very important for starvation-induced activation of the PIK3C3 lipid kinase in the PtdIns3K-C1 complex [25,27]. Strikingly, the level of ATG14 phosphorylated at S29 was strongly reduced for the LIR mutant both in basal conditions and upon 6 h starvation (Figure 7(c,e)). Phosphorylation of BECN1 at S93 was also increased after 6 h of starvation, but was not dependent on the ATG14 LIR interaction (Figure 7(c,f,g)). Taken together, this shows that the LIR mutant of ATG14 does not impair PtdIns3K-C1 complex formation, but is required for efficient phosphorylation of ATG14 S29, contributing to the formation of an active PtdIns3K-C1 complex.

### The ATG14 LIR is important for colocalization with LC3B and efficient mitophagy

**Figure 8.**
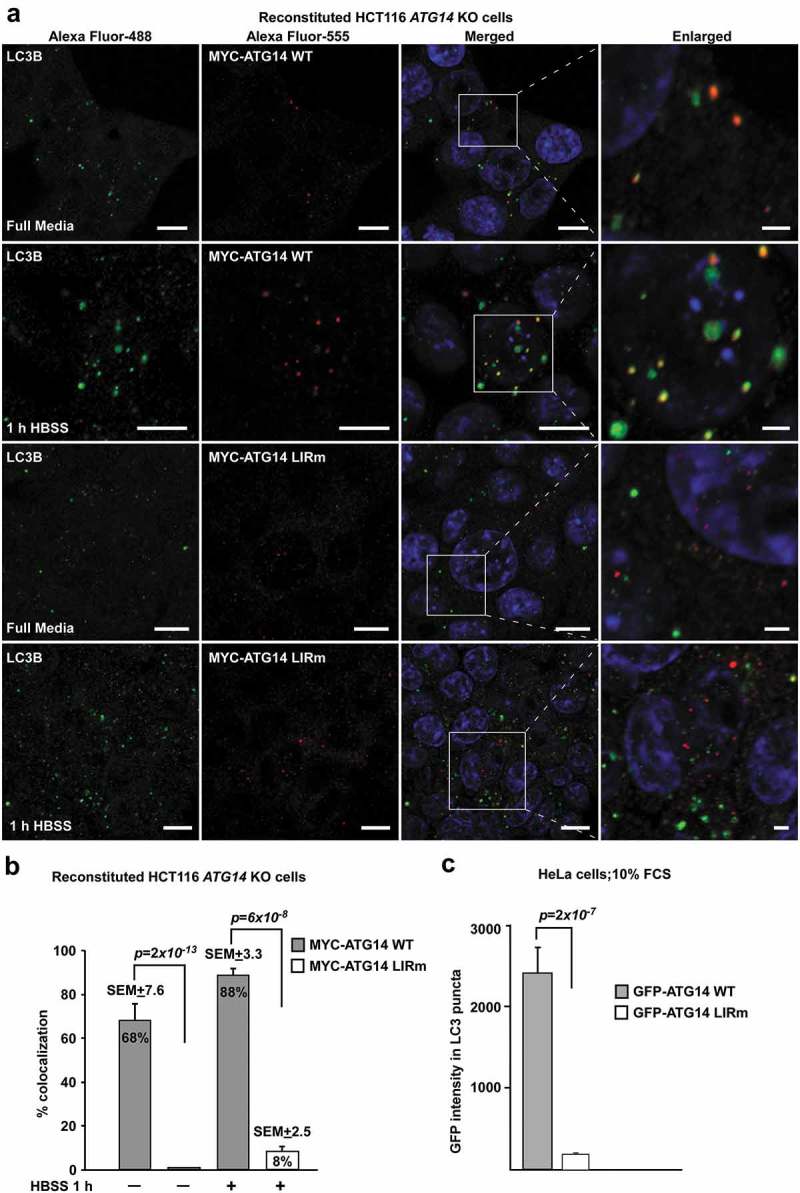
The ATG14 LIR interaction is important for colocalization with LC3B. (**a**) HCT116 cells knocked out for *ATG14* and reconstituted with WT MYC-ATG14 (upper panels) or LIR mutated MYC-ATG14 (lower panels) were immunostained with MYC and LC3B antibodies and analyzed by confocal imaging. Scale bars: 10 µm and 2 µm (Enlarged). (**b**) Quantification of the colocalization analyzed in 8A, represented as percentage of Myc-ATG14 dots also containing LC3B. The quantification is based on analysis of 100–150 cells. (**c**) Quantification of EGFP intensity inside cytosolic LC3 puncta in 110–180 HeLa cells transfected with EGFP-ATG14 WT or LIR mutant. Data information: Means ± SEM; *n* ≥ 100 cells. Significant *P* values are indicated (Student´s two-tailed, unpaired *t*-test).

**Figure 9.**
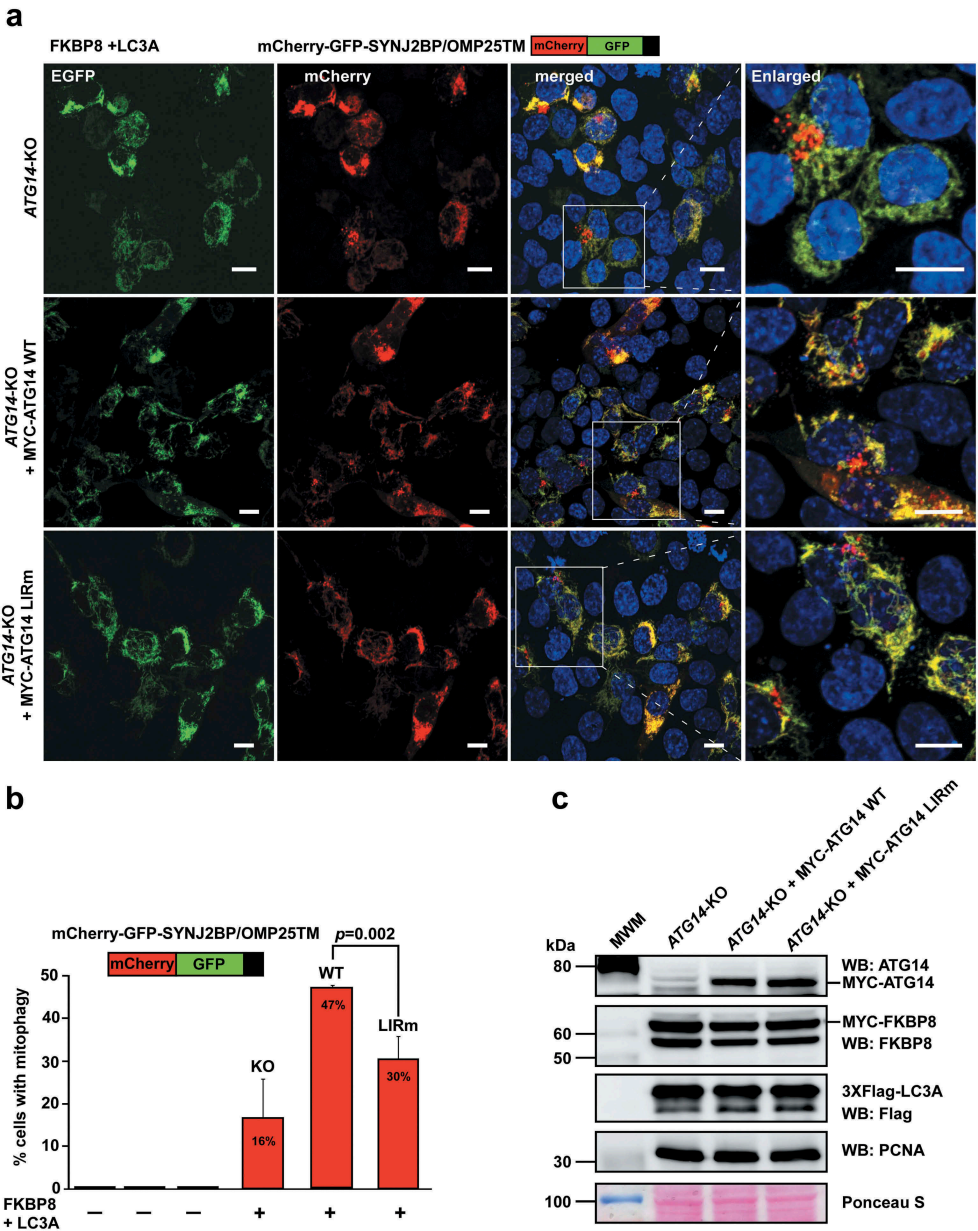
The ATG14 LIR interaction is important for efficient mitophagy induced by the co-expression of FKBP8 and LC3A. (**a**) HCT116 *ATG14* KO cells reconstituted or not with WT or LIR mutated MYC-ATG14 were transiently transfected with FKBP8 and LC3A, in order to induce mitophagy, along with the mitophagy marker SYNJ2BP/OMP25-TM fused to the mCherry-GFP double tag. Mitophagy was then measured as the appearance of red-only structures. Scale bars: 10 µm. (**b**) Quantification of the data analyzed in 9A. The graph bars represent quantification of cells containing red‐only dots in cells expressing mCherry‐GFP‐OMP25‐TM, done manually in 60–100 cells in 3 independent experiments. Each bar shows the mean value with standard error of the mean (SEM). Two-tailed, unpaired student t-test was used to calculate p-values. (**c**) Western blots showing equal levels of MYC-FKBP8 and 3xFlag-LC3A expressed in the 3 cell lines. Data information: Data represent mean ± SD of 3 independent experiments (60–100 cells analyzed manually for each cell type for each experiment). Significant *P* values are indicated (Student´s two-tailed, unpaired *t*-test).

As previously described [44], the *ATG14* KO HCT116 cells displayed defective autophagy, with accumulation of SQSTM1 and LC3B form I and II under basal and starvation conditions (Fig. S6B and C). Reconstitution with MYC-ATG14 WT or MYC-ATG14 LIR mutant rescued the KO phenotype under different conditions as analyzed by western blots for lipidation of LC3B and GABARAP as well as by degradation of SQSTM1, NBR1, CALCOCO2/NDP52 and TAX1BP1 (Fig. S6D and E). We then tested the importance of the ATG14 LIR motif for colocalization with phagophores by monitoring the colocalization of MYC-ATG14 and endogenous LC3B puncta under normal growth conditions as well as during starvation. Strikingly, the ATG14 LIR mutant puncta did not colocalize with LC3B puncta, demonstrating a clear LIR-dependence for recruitment of ATG14 to phagophores (Figure 8(a,b)). Analysis of endogenous LC3B colocalization of overexpressed EGFP-ATG14 and EGFP-ATG14 LIR mutant in HeLa cells revealed the same LIR dependency (Figure 8(c)). Moreover, analysis of LAMP1-positive SQSTM1 puncta in the reconstituted *ATG14* KO cells during 6 h starvation in the presence of lysosomal inhibition (BafA1), revealed a significant decrease of SQSTM1 puncta that were LAMP1-positive for the LIR mutant reconstituted cells compared to the WT (Fig. S7A and B). This indicates that the ATG14 LIR motif is also important for efficient sequestration of SQSTM1 into late endosome/lysosomal structures. To further investigate the functional impact of the ATG14 LIR mutation on a selective autophagy process where there is demand for generation of many autophagosomes we turned to mitophagy. We took advantage of our recent finding that mitophagy is very effectively induced by transient overexpression of FKBP8 and LC3A and monitored mitophagy using mCherry-EGFP-SYNJ2BP/OMP25-TM [50]. The latter acts as a reporter of mitochondria sequestered into acidic autolysosomes where they appear as red only dots. We observed a significant rescue of mitophagy in the reconstituted cells, compared to the *ATG14* KO cell line. However, the ATG14 LIR mutant was unable to rescue the mitophagy as efficiently as the WT and displayed a 37% reduction in mitophagy relative to the WT (Figure 9(a–c)).

## Discussion

We previously identified LIR motifs in components of the mammalian ULK1/2 complex [39]. ULK1, ULK2, ATG13, and RB1CC1/FIP200 all contain LIR motifs preferentially interacting with the GABARAP subfamily. These interactions facilitate and/or stabilize association of the ULK1/2 complex on the phagophore. In this study, we identified single functional LIR motifs in PIK3C3, BECN1 and ATG14 of the autophagy-specific PtdIns3K complex I which all display a preference for binding to GABARAP and GABARAPL1. We obtained high-resolution crystal structures of chimeric proteins consisting of GABARAP N-terminally fused to the LIR motifs of PIK3C3 and BECN1 with a Gly-Ser linker, and LIR peptides from BECN1 and ATG14 co-crystallized with GABARAPL1. The 6 structures provide insights into amino acid specificity preferences of the LIR motifs as seen in 2D peptide array scans and provide an explanation for the binding preference towards GABARAP and GABARAPL1. To assess the functional importance of LIR motifs in the complex we studied the effects of mutating the LIR motifs of ATG14, the sole autophagy-specific component of the complex. Although the LIR was not required for formation of the PtdIns3K complex I, it was crucial for colocalization with LC3B. Furthermore, the LIR motif was required for phosphorylation of ATG14 S29 suggesting that the LIR motif may facilitate activation of the complex by ULK1. Finally, the ATG14 LIR was needed for efficient FKBP8- and LC3A-induced mitophagy.

The importance of each position of the LIR motifs of PIK3C3, BECN1 and ATG14 for binding to GABARAPL1 was tested using 2D peptide array scans (Figure 3). The LIR-F250 of PIK3C3 (FELV) is a canonical F-type LIR motif with some stringent requirements for which substitutions were allowed in positions +2 and +3 and a requirement for D or E at position −1. The LIR motif of BECN1 (FTLI) is also a canonical F-type LIR motif. It displayed an even higher sensitivity towards substitutions in all the 4 core LIR motif positions than seen for the PIK3C3 LIR and other analyzed LIR motifs. The ATG14 LIR-W435 (WENL) on the other hand, is a W-type LIR motif with a clear preference for D or E in position −1 and displayed almost complete flexibility for substitutions at the +2 position. The ATG14 LIR bound most strongly with Kds of 2.85 μM and 1.9 μM for binding to GABARAP and GABARAPL1, respectively, followed by BECN1 (Kds of 10.5 μM and 7.95 μM), while PIK3C3 bound more weakly with Kds of 49.5 μM and 65 μM (Table 1). Using phosphomimetic mutants of known phosphorylation sites preceding the BECN1 and PIK3C3 LIRs we revealed a marked increased affinity for binding of BECN1 to GABARAP, GABARAPL1 and LC3C with a striking increase in affinity of the phosphomimetic mutations at S244 and S249 for PIK3C3 of up to 19-fold. This implies that the LIR motifs of BECN1 and PIK3C3 might be regulated by phosphorylation. Several known LIR-Atg8-family protein interactions are modulated by phosphorylation [30]. Known phosphorylation events regulating PtdIns3K-C1 converge on the BECN1 and ATG14 N-terminal domains as well as on the linker region between the PIK3C3 C2 domain and the HELCAT (helicase-catalytic) domain. AMPK (5ʹ-AMP-activated protein kinase) activates pro-autophagy complexes (containing ATG14) by phosphorylating BECN1 at S93, S96 preceding the LIR [45]. Interestingly, we found the corresponding phosphomimetic mutants to bind with higher affinity to GABARAP and GABARAPL1. The presence of ATG14 in the complex inhibits the phosphorylation on PIK3C3 and increases phosphorylation of BECN1 by AMPK [45]. ATG14-dependent phosphorylation of BECN1 at S90 has also been demonstrated [44]. The stress-responsive kinases MAPKAPK2 (MAP kinase-activated protein kinase 2) and MAPKAPK3 phosphorylate S90 of BECN1 increasing the lipid kinase activity of the BECN1-PIK3C3 complex [51]. DAPK3 (death associated protein kinase 3) directly phosphorylates BECN1 at S90 and PPP2/protein phosphatase 2A is implicated in regulation of BECN1 S90 phosphorylation [52]. The ULK1 kinase phosphorylates PIK3C3 at S249 (preceding the LIR in the linker region) and S96 in BECN1 is a predicted ULK1 phosphorylation site [47]. Our structure of the phosphomimetic S96E mutant of BECN1 LIR peptide bound to GABARAP revealed an additional water-bridged hydrogen bond between S96E and K46 in GABARAP (Figure 5(b)). This may partly explain the observed increased binding of the BECN1 phosphomimetic mutant to GABARAP (Figure 2). Similarly, the phosphomimetic S249E mutant of PIK3C3 interacted with K48 (GABARAP) via a salt bridge (Figure 4(b)). The S249E mutant LIR peptide bound much more strongly to GABARAP, GABARAPL1 and LC3C, and even bound significantly to LC3B (Fig. S1B). Thus, *in vivo* phosphorylation of S249 may enhance the LIR interaction in PIK3C3.

The binding preference for a LIR motif may partly be determined by the sequence of the core LIR motif. The sequence [W/F]-[V/I]-x-V named GABARAP interaction motif (GIM) was recently proposed as a consensus for core LIR sequences mediating selective binding to GABARAPs [53]. However, the core LIR motifs of autophagy-specific PtdIns3K-C1 proteins do not fit into this consensus motif. The solved crystal structures indicated that the binding preference for the GABARAP subfamily can be explained by hydrophobic interactions between the residues C-terminal to the core LIR motif in position +4 to +6 and the hydrophobic pocket 2 (Fig. S5). Residues F60 and L55 of GABARAP hydrophobic pocket 2 interacted with position +3 as well as position +4 to +6 of the LIR motifs and are conserved in GABARAPL1, GABARAPL2 and LC3C. In LC3A/B the F60 and L55 residues are replaced by shorter residues (L63 and V58), potentially explaining reduced interactions. Furthermore, D54 is replaced by H57 in LC3B and clashed with the residues in position +4 to +6 of each LIR motif. Thus, we propose that these 3 conserved residues of hydrophobic pocket 2 mediate the specificity of the PtdIns3K-C1 LIR motifs. Similar interactions have been identified in other autophagy mediating proteins containing LIR motifs. The specificity of the LIR motif of WDFY3/ALFY for GABARAP is partly mediated by an interaction between D54 of GABARAP and a tyrosine residue C-terminal to the core LIR motif [48]. For FYCO1, H57 of LC3B interacts with an aspartate residue in position +5 C-terminal to the core LIR motif (corresponding to the tyrosine residue in WDFY3) providing specificity towards binding to LC3A/B [49].

With the LIR motifs in the autophagy-specific PtdIns3K complex I identified in this study, the list of LIR motifs in the basal autophagy apparatus is expanding (Figure 3(d)). The components of the mammalian ULK1/2 complex, ULK1, ULK2, ATG13, and RB1CC1 all contain canonical F-type LIR motifs preferentially interacting with the GABARAP subfamily. Consistently, *C. elegans* UNC-51 (Atg1/ULK1 ortholog) interacts with the GABARAP-like Atg8 homolog LGG-1, but not with the LC3 homolog LGG-2 [54]. This binding preference towards the GABARAP subfamily by 2 of the important core complexes involved in autophagy initiation is intriguing. Why do the core machinery components preferentially bind to GABARAP family proteins? Yeast and other fungi have only a single Atg8 homolog whereas *Drosophil*a has 2. These are most similar to vertebrate GABARAP [28]. Macroautophagic bulk sequestration in hepatocytes and prostate cancer cells is independent on the LC3 subfamily, but requires the GABARAP subfamily [55]. Using a knockout strategy in HeLa cells, members of the GABARAP subfamily were identified as the main contributors to mitophagy and crucial for autophagosome–lysosome fusion [56]. PLEKHM1 (pleckstrin homology domain-containing family M member 1), which regulates autophagosome-lysosome fusion, is also preferentially interacting with GABARAPs [57]. Knockdown of GABARAP inhibits ULK1 activation, and WAC (WW domain containing adaptor with coiled-coil) acts as a positive regulator of autophagy by releasing unlipidated GABARAP from the centrosome. GABARAP is then subsequently transported to autophagosome formation sites [58]. Like GABARAP, ATG14 is involved in both early and late steps of autophagy [14,59].

Our data from reconstituted HCT116 *ATG14* KO cells showed that the efficient binding of BECN1 and PIK3C3 to GABARAP and GABARAPL1, unlike binding of ULK1, depends on the ATG14 LIR interaction (Figure 7(a)). The ATG14 LIR-GABARAP or ATG14 LIR-GABARAPL1 interaction is clearly stronger than the corresponding interactions with the BECN1 and PIK3C3 LIRs. The ATG14 LIR is also the one that most likely is bound to Atg8-family proteins on the phagophore in the assembled PtdIns3K-C1 (Figure 6(b)). Similarly to the ULK1 LIR mutant [39], the ATG14 LIR mutant did not colocalize with LC3. We speculate that the LIR-Atg8-family protein interaction helps keep ULK1 and ATG14 stably attached to the phagophore while the LIR mutants are only capable of transient interactions. The ATG14 LIR interaction was important for efficient phosphorylation of S29 of ATG14, seen both under basal and starvation conditions (Figure 7(c,e)). Phosphorylation of ATG14 S29 by ULK1/2 is crucial for the activation of the lipid kinase activity of the complex [25,27]. LIR-dependent conformational changes in ATG14 could allow ULK1/2 to access its target site. In the same or an alternative scenario, the ATG14 LIR interaction enables tethering of the whole complex at the phagophore. This would allow proximity to the ULK1/2 complex (already present at the phagophore) and thus efficient phosphorylation. ULK1/2 not only phosphorylates ATG14 but also other complex members as mentioned above, and acts as a key player in the regulation of the PtdIns3K complex I. Interestingly, our previous investigations on the LIR interactions of the ULK1/2 complex also support GABARAP mediated scaffolding of the ULK1/2 complex at the phagophore [39]. The cumulative effect of the GABARAP-LIR interactions of these two core autophagy complexes could be concentration of the interacting complexes at the expanding autophagosome.

The C-terminal 80 amino acids BATS domain contains an amphipathic alpha helix belonging to a class of ALPS (amphipathic lipid packing sensor) motifs that binds directly to curved membranes and is sufficient to target ATG14 to autophagosomes [15]. The BATS domain binds both PtdIns3P and PtdIns(4,5)P_2_ [15,24]. Mitochondrial outer membranes contain PtdIns(4,5)P_2_ and ATG14 is recruited to ER-mitochondria contact sites where autophagosomes form [60]. The membrane-scaffolding role of Atg8-family protein-LIR interactions may in many cases be involved in targeting of the proteins in question to specific membranes through coincidence detection mechanisms. The LIR motif in ATG14 is located about 30 amino acids N-terminal to the ALPS motif in the BATS domain. Together, these two motifs may enable coincidence detection for specific targeting of the phagophore and perhaps also the mature autophagosome because ATG14 is also involved in fusion of the autophagosome with the lysosome [14]. The combination of LIR motifs and membrane-binding domains for coincidence detection is likely a more general feature for a number of vesicle-associated LIR-containing proteins, including members of the autophagy core machinery like ATG14, PIK3C3, BECN1, ULK1/2, ATG13, ATG3, and other proteins associated with autophagosomes and/or endosomes like FYCO1 and PLEKHM1 [57,59,61,62].

Recent advances in structural information have provided us with a picture of the V- or Y-shaped PtdIns3K complex I as a dynamic bipartite structure with catalytic activity in the PIK3R4-PIK3C3 arm and functional specificity arising from the BECN1-ATG14 arm and base [6,16]. PIK3R4, which does not interact with Atg8-family proteins, acts as a bridge between the BECN1-ATG14 arm and the PIK3C3 lipid kinase. PIK3R4 functions as an allosteric brake. The active state of the complex involves dislodging of the PIK3C3 catalytic subunit [63]. Interestingly, the LIR motif of PIK3C3 is located at the start of the long linker region (Figures 1(a) and 6(b)) that is predicted to be crucial for enabling the HELCAT domain to reach its lipid substrates [63]. The LIR interactions of the PtdIns3K complex I could be involved in facilitating the dislodging of the HELCAT domain leading to an active complex conformation. Future structural studies at even higher resolution are needed to reveal how the interplay between GABARAP proteins and LIRs in the PtdIns3K-C1 complex is acting together with the other membrane-binding- and membrane curvature-sensing domains. Such studies will shed light on contributions of LIR interactions in forming the active state of the complex.

## Materials and methods

### Antibodies

The following primary antibodies were used: HRP-conjugated anti-GST (GE Healthcare, RPN1236), rabbit polyclonal anti-GFP (Abcam, ab290), rabbit polyclonal anti-LC3B (Sigma, L7543); mouse monoclonal anti-LC3B (Nano tools, 0231–100/lc3-5F10), mouse monoclonal anti-GABARAP (MBL, M153-3), mouse monoclonal anti-WIPI2 (Abcam, ab105459), mouse monoclonal anti-MYC-9B11 clone (Cell Signaling Technology, 2276), rabbit polyclonal anti-ATG14 (Cell Signaling Technology, 5504), mouse monoclonal anti-BECN1 (BD Bioscience, 610153), rabbit polyclonal phosphor-specific Ser93-BECN1 D9A5G (Cell Signaling Technology, 14717), rabbit polyclonal phosphor-specific Ser29-ATG14 (Cell Signaling Technology, 13155), rabbit monoclonal anti-PtdIns 3-kinase class III/PIK3C3-D4E2 (Cell Signaling Technology, 3358), mouse monoclonal anti-PIK3R4 (Abnova, H00030849-M02), mouse monoclonal anti-SQSTM1/p62-lck ligand (BD Bioscience, 610833), guinea pig polyclonal anti-SQSTM1/p62 (Progen, GP62-C), mouse monoclonal anti-LAMP1 (DSHB, H4A3), mouse monoclonal anti-NBR1 (Santa Cruz Biotechnology, sc-130380), rabbit polyclonal anti-TAX1BP1 (Sigma, HPA024432), mouse monoclonal anti-PCNA (DAKO, M0879), rabbit polyclonal anti-CALCOCO2 (Sigma, HPA023195), mouse monoclonal anti-FLAG M2 antibody (Sigma, F1804), rabbit monoclonal anti-FKBP8 (Abcam, ab129113). Secondary antibodies: HRP (horseradish peroxidase)-conjugated goat anti-rabbit IgG (BD Bioscience Pharmingen, 554021) and HRP-conjugated anti-Biotin antibody (Cell Signaling Technology, 7075). Following fluorescent secondary antibodies were used: Alexa Fluor® 488‐conjugated goat anti‐rabbit IgG (Life Technologies, A-11,008), Alexa Fluor® 488‐conjugated goat anti‐mouse IgG (Life Technologies, A-11,029), Alexa Fluor® 555-conjugated goat anti-mouse IgG (Life Technologies, A-21422), Alexa Fluor® 555-conjugated goat anti-rabbit IgG (Life Technologies, A-21428), Alexa Fluor® 647‐conjugated goat anti‐mouse IgG (Life Technologies, A21236) and Alexa Fluor® 647‐conjugated goat anti‐guinea pig IgG (Life Technologies, A21450).

### Plasmids

Plasmids used in this work are listed below. Point mutations were generated using the QuikChange site-directed mutagenesis kit (Stratagene, 200518) and Gateway destination plasmids were made using Gateway LR recombination reactions (Invitrogen, 11791100) following the manufacturer’s instructions. All plasmid constructs made in this study were verified by DNA sequencing (BigDye sequencing kit, Applied Biosystems, 4337455). The oligonucleotides used for mutagenesis, PCR, and DNA sequencing were purchased from Invitrogen.

### Gateway cloning vectors

**Table:** 

Plasmid	Description	Source
pDEST-MYC	mammalian MYC-tag fusion expression vector, *CMV* and *T7* promoters	[64]
pDest-EGFP-C1	mammalian EGFP fusion expression vector, CMV promoter	[64]
pENTR1A	Gateway entry vector	Invitrogen, 410462
pDONR221	Gateway donor vector	Invitrogen, 12536017
pDONR223	Gateway donor vector	Addgene, 23457

### Gateway entry clones

**Table:** 

Plasmid	Source
pDONR223-PIK3C3 (PIK3C3)	Addgene, 23457
pDONR223-PIK3C3 F198A/V201A	This study
pDONR223-PIK3C3 F250A/V253A	This study
pDONR223-PIK3C3 F198A/V201A/F250A/V253A	This study
pENTR-BECN1	This study
pENTR-BECN1 F97A/I100A	This study
pENTR-BECN1 S90E/S93E/S96E	This study
pENTR-BECN1 F97A/I100A S90E/S93E/S96E	This study
pDONR221-ATG14	This study
pDONR221-ATG14F64A/F67A	This study
pDONR221-ATG14W435A/L438A	This study
pDONR221-ATG14F64A/F67A/W435A/L438A	This study

### Gateway expression clones

**Table:** 

Plasmid	Source
pDest15 (GST)-LC3A	[33]
pDest15 (GST)-LC3B	[33]
pDest15 (GST)-LC3C	[39]
pDest15 (GST)-GABARAP	[33]
pDest15 (GST)-GABARAPL1	[33]
pDest15 (GST)-GABARAPL2	[33]
pDest-MYC-PIK3C3	This study
pDest-MYC-PIK3C3 F198A/V201A	This study
pDest-MYC-PIK3C3 F250A/V253A	This study
pDest-MYC-PIK3C3 F198A/V201A/F250A/V253A	This study
pDest-MYC-BECN1	This study
pDest-MYC-BECN1 F97A/I100A	This study
pDest-MYC-BECN1 S90E/S93E/S96E	This study
pDest-MYC BECN1 F97A/I100A/S90E/S93E/S96E	This study
pDest-MYC-ATG14	This study
pDest-MYC-ATG14 F65A/F67A	This study
pDest-MYC-ATG14 W435A/L438A	This study
pDest-MYC-ATG14 F65A/F67A/W435A/L438A	This study
pDest-EGFP-ATG14	This study
pDest-EGFP-ATG14 W435A/L438A	This study
pDest-MYC-FKBP8	[50]
pDest-3X-Flag-LC3A	[50]

### Retroviral expression vectors

**Table:** 

Plasmid	Description	Source
pMX-Puro	Retroviral vector with puromycin selection marker	Cell Biolabs Inc, RTV-012
pMX-Puro-MYC-ATG14-WT	Retroviral vector with puromycin selection marker encoding full length ATG14-WT	This study
pMX-Puro-MYC-ATG14-LIRm	Retroviral vector with puromycin selection marker encoding full length ATG14-LIRm	This study

### Cell cultures and transfections

HCT116 cells were grown in McCoy’s 5A media (Sigma, M9309) supplemented with 10% fetal bovine serum (Biochrom AG, S0615). HeLa cells were grown in Eagle’s minimum essential medium (Sigma, M4655) supplemented with 10% fetal bovine serum, non-essential amino acids, 2 mM L-glutamine, and 1% streptomycin-penicillin (Sigma, P4333). HEK293-Phoenix packaging cells were cultured in Dulbecco’s modified Eagle’s medium with the same supplements as described above for HeLa cells. For starvation, cells were incubated in Hanks Balanced Salt Solution (HBSS; Sigma, H8264) for 1, 4 or 6 h. Cells were treated as indicated with 0.2 μM bafilomycin A_1_ (Sigma, B1793). Subconfluent cells were transfected with the indicated plasmids using TransIT-LT1 (Mirus, MIR2300) or MetafectenePro (Biontex, T040) following the supplier’s instructions. Approximately 24 h after transfection, HCT116 cells were fixed in 4% paraformaldehyde (PFA) prior to processing for image analysis. Notably for fluorescence microscopy analysis of mitochondria, HCT116 cells were fixed with 4% PFA at 37°C. 12 to 14 h after transfection, HeLa cells were fixed in 4% PFA.

### Reconstitution of HCT116 ATG 14L KO cells for stable expression of MYC-tagged ATG14

Full-length MYC-tagged ATG14-WT/LIRm was amplified with PCR and cloned into *Eco*RI and *Not*I restriction sites of the retroviral expression vector pMX-Puro (Cell Biolabs Inc, RTV-012) with the selection marker puromycin. The HEK293-Phoenix packaging cell line (ATCC, CRL-3213) was transfected with pMX-Puro-Myc-ATG14-WT/LIRm expression vector using MetafectenePro. The virus containing media from transfected HEK293-Phoenix cells was harvested 24, 48 and 72 h post transfection. The harvested media was subsequently filtered through a 0.45-µm filter and then added onto subconfluent HCT116 *ATG14* KO cells (a geneorus gift of R. Youle, National Institutes of Health, Bethesda, MD, USA) [44]. Hexadimetrine bromide/polybrene (Sigma, H9268) was then added to a final concentration of 8 µg/ml. The HCT116-*ATG14*-KO cells were incubated with the virus-containing media with polybrene for 6–12 h each time. The transduced HCT116 *ATG14* KO cells were then selected with 1 µg/ml of puromycin (InvivoGen, ant-pr-1). Stable expression of MYC-ATG14-WT/LIRm was verified by western blotting using anti-MYC antibody.

### GST affinity-isolation assays

GST affinity-isolation assays were performed by incubating immobilized GST or GST-tagged proteins with ^35^S-labeled *in vitro*-translated proteins or total cell lysate from HCT116-*ATG14* KO cell line reconstituted with MYC-ATG14-WT/LIRm. All GST-tagged proteins were expressed in *Escherichia coli* SoluBL21 (Genlantis, C700200). GST fusion proteins were purified on glutathione-Sepharose 4 Fast Flow beads (GE Healthcare, 17–5132-01). ^35^S-labeled MYC-tagged proteins were synthesized *in vitro* using the TNT T7-coupled reticulocyte lysate system (Promega, L4610). Diluted *in vitro* translated proteins or total cell lysates were pre-cleared by incubation with glutathione-coupled Sepharose beads for 30 min prior to incubation with immobilized GST or GST fusion proteins for 2 h at 4°C in NETN buffer (50 mM Tris [VWR, 28811.295], pH 8.0, 100 mM NaCl [Sigma, 71380], 1 mM EDTA [Sigma, 34549], 0.5% Nonidet P-40 [Sigma, 74385]) supplemented with cOmplete Mini EDTA-free protease inhibitor mixture tablets (1 tablet/10 ml) (Roche Applied Science, 11836170001). The beads were washed 5 times with NETN buffer to remove unbound proteins. Bound proteins were then eluted by boiling in 2× SDS-PAGE gel loading buffer (125 mM Tris, pH 7.5, 4% SDS (Sigma, 05030), 0.04% bromophenol blue, 8% sucrose, 100 mM dithiothreitol) with 1 mM DTT [Sigma, D0632], and subjected to SDS-PAGE. Gels were stained with Coomassie Brilliant Blue (Thermo Scientific, 20278) and vacuum-dried. ^35^S-labeled proteins were detected using a Fujifilm bioimaging analyzer BAS-5000 (Fuji). Signals from ^35^S-labelled proteins were measured in terms of unit of photostimulated luminescent (PSL) and quantified in comparison with 10% of the *in vitro* translated lysate using the Image Gauge software (Fuji) [65]. For GST affinity isolation with lysates from HCT116 cells, subconfluent HCT116 *ATG14*-KO cells reconstituted with retroviral based MYC-ATG14-WT or LIRm were rinsed with ice-cold PBS (Gibco, 70011–036) prior to lysis in ice-cold RIPA buffer (50 mM Tris, pH 7.5, 150 mM NaCl, 1mM EDTA, 1% NP40 (Sigma, 74385), 0.25% Triton X-100 (VWR, 28817.295) supplemented with cOmplete Mini EDTA-free protease inhibitor mixture for 30 min on ice. Cell lysates were incubated overnight with GST or GST-bound protein immobilized on beads. Beads were then washed 5 times with RIPA buffer, and bound protein was eluted by boiling in 2X SDS gel loading buffer and subsequently subjected to SDS-PAGE. GST or GST bound proteins were visualized by Ponceau (Sigma, P3504) staining and bound proteins were visualized by immunoblotting using specific antibodies

### Immunostaining and confocal microscopy

For immunofluorescent staining, fixed cells were permeabilized with pre-chilled (−20°C) methanol. The permeabilized cells were blocked with 3% pre-immune goat serum (Sigma, G6767) in PBS for 1 h at room temperature before incubation for 2 h at room temperature with primary antibodies diluted in PBS with 1% goat serum. Cells were then incubated for 1 h with Alexa Fluor® secondary antibodies diluted 1:500 (or 1:5000) in PBS supplemented with 1% goat serum. Cells were imaged using an LSM780 confocal microscope (Carl Zeiss Microscopy) equipped with a 40X NA1.2 water immersion lens and 63X NA1.4 plan-apochromat objective. Fluorescence channels were configured using SmartSetup in ZEN 2012 (black edition), eliminating spectral bleed through. For image analysis, random image fields were recorded for each experimental condition, using identical, non-saturating scan settings. Images were analyzed in Volocity ver. 6.3 (PerkinElmer), using a custom-built protocol.

### Immunoblots and immunoprecipitation

The cell extracts of HCT116 WT cells, HCT116 *ATG14* KO and reconstituted MYC-ATG14-WT/LIRm were resolved by SDS-PAGE and transferred to Hybond-ECL nitrocellulose membrane (GE healthcare, GE10600003). Transfer was visualized with Ponceau staining and the membrane was blocked with 5% non-fat dry milk in TBST (20 mM Tris pH 7.5, 150 mM NaCl, 0.1% Tween 20 [Sigma, P1379]). The membrane was incubated with primary antibody overnight at 4°C followed by incubation with HRP-conjugated secondary antibody for 1 h at room temperature. Signal detection was performed with a western blotting chemiluminescent reagent (Sigma, CPS3500) and a LumiAnalyst imager (Roche Applied Sciences). For MYC-Trap: subconfluent HCT116-*ATG14*-KO and/or reconstituted MYC-ATG14-WT/LIRm cells supplied with full media or HBSS for 6 h, rinsed with PBS, lysed with ice cold CHAPS buffer (40 mM HEPES [Sigma, H7006], pH 7.5, 120 mM NaCl, 1 mM EDTA, 1% CHAPS [Sigma, C3023]) supplemented with cOmplete Mini EDTA-free protease inhibitor mixture and phosphatase inhibitor cocktail. Cells were scraped and the collected lysate was passed through 25G and 21G needles 30–40 times each before centrifugation at 16,000 x g for 10 min. The resultant supernatant was incubated with MYC-Trap beads (Chromotek, yta20) overnight. The precipitated immunocomplex was washed 5 times with CHAPS buffer and the proteins eluted by boiling for 5 min in 2xSDS gel loading buffer.

### SPOT synthesis of peptide arrays and GST overlay assay

PIK3C3, BECN1 and ATG14 peptide arrays were synthesized on cellulose membranes using MultiPrep peptide synthesizer (INTAVIS Bioanalytical Instruments AG, Germany). Membranes were blocked with 5% of non-fat milk in TBST and peptide interactions were tested with GST-GABARAP or GST-GABARAPL1 by overlaying the membrane with 1 µg/ml of recombinant protein and incubating for 2 h at room temperature. Bound proteins were visualized with HRP-conjugated anti-GST antibody [65]. Putative LIR motifs in 20, 3 arrays (20-mer peptides moved a window of 3 residues along the protein sequence) were identified as 4–6 consecutive strong spots containing the core LIR consensus (W/F/Y)XX(L/I/V).

### Protein expression and purification for crystallization

GABARAPL1 full-length or GABARAP chimera sequences were inserted between the *Bam*HI and *Not*I sites of a pGEX-6P2 plasmid (GE Healthcare, 28–9546-50) containing a cleavable glutathione S-transferase tag. Protein expression was performed at 30°C in *E. coli* Rosetta (DE3) pLysS (Novagen, 71403). Bacteria were harvested by centrifugation and resuspended in lysis buffer (50 mM Tris-HCl, pH 8.0, 500 mM NaCl, 0.1% TX-100, 0.5 mM TCEP [Melford Laboratories, T2650], 0.5 mM AEBSF [AppliChem, A1421], 15 μg/ml benzamidine [Melford Laboratories, B4101]). The fusion protein was batch-adsorbed onto a glutathione-Sepharose affinity matrix and GABARAPL1 recovered by cleavage with 3C protease (made in house) at 4°C overnight in 50 mM Tris-HCl, pH 8.0, 100 mM NaCl, 0.5 mM TCEP. The protein was then purified by size exclusion chromatography using a Superdex 75 column equilibrated and run in 25 mM Tris-HCl, pH 8.0, 150 mM NaCl, 0.5 mM TCEP. Peptides were synthesized by the Francis Crick Institute Peptide Chemistry Science Technology Platform.

### Crystallisation and data processing

GABARAPL1-peptide LIR complexes were prepared by mixing purified full-length GABARAPL1 and BECN1 peptide (residues 93–102, SANSFTLIGE) or ATG14 peptide (residues 429–443, TDLGTDWENLPSPRF) at a 1:3 molar ratio. The complexes were dialyzed overnight in 25 mM Tris-HCl, pH 8.0, 150 mM NaCl, 0.5 mM TCEP buffer, using a 500–1000 Da MWCO dialysis tubing (Sigma, D0405) for both complexes. All the peptide-GABARAPL1 complexes or chimeras were crystallized at 20°C using the sitting-drop vapor diffusion method with a protein concentration of 10–20 mg/ml. Initial crystallization trial was peformed using Qiagen (JCSG core 1–4, AMSO4), Molecular dimension (PACT, Wizard 1–4), Jena Bioscience (PiPEG). In all cases the drop included 0.5 μl of protein and 0.5 μl of mother liquor. For WT PIK3C3[244–258]-GABARAP crystals grew in 50 mM HEPES, pH 7.1, 40% PEG 600. For S249E PIK3C3[244–258]-GABARAP crystals grew in 20% PEG 3350 (Hampton Research, HR2-591), 200 mM magnesium formate (Sigma, 86301). For WT BECN1[93–105]-GABARAP crystals grew in 15% PEG 4000 (Sigma, 807490), 50 mM Bicine (Sigma, B3876), pH 8.8. For S96E BECN1[93–105]-GABARAP crystals grew in 1.6 M ammonium sulfate (Hampton Research, HR2–541), 100 mM Tris pH 8.5, 10% glycerol. For BECN1 LIR peptide-GABARAPL1 crystals grew in 100 mM sodium acetate, 100 mM HEPES, pH 7.5, 12% PEG 4000. For ATG14 LIR peptide-GABARAPL1 crystals grew in 1 M lithium chloride, 100 mM HEPES pH 7, 20% PEG 6000. Crystals were flash-frozen in liquid nitrogen, and X-ray data sets were collected at 100 K at the I03, I04 and I24 beamline of the Diamond Light Source Synchrotron (Oxford, UK). Data collection and refinement statistics are summarized in Table 2. The data sets were indexed and scaled with xia2 [66]. Molecular replacement was achieved by using the atomic coordinates of the peptide-free GABARAPL1 (PDB code: 2R2Q) and GABARAP (PDB code: 1GNU) in PHASER [67]. Refinement was carried out using Phenix [68]. Model building was carried out in COOT [69]. Model validation used PROCHECK [70], and figures were prepared using the graphics program PYMOL (http://www.pymol.org).

### Bio-layer interferometry assay

Bio-layer interferometry (BLI) is an optical analytical technique for measuring kinetics of interactions in real-time. The biosensor tip surface immobilized with a ligand is incubated with an analyte in solution, resulting in an increase in optical thickness at the biosensor tip and a wavelength shift, which is a direct measure of the change in thickness. Biolayer interferometry analysis of Atg8-family proteins binding to immobilized biotinylated-LIR peptides were studied using an Octet Red 96 (ForteBio). 50 μg/ml of biotin-LIR peptide were immobilized on streptavidin coated biosensor (SA, ForteBio) and the typical immobilization levels were above 0.15 nm. Ligands-loaded SA biosensors were then incubated with different concentrations of Atg8-family protein. Global fitting of the binding curves generated a best fit with the 1:1 model and the kinetic association and dissociation constants were calculated. All binding experiments were performed in solid-black 96-well plates containing 200 μl of solution in each well at 25°C with an agitation speed of 1000 rpm.

### Statistical analyzes

Data are expressed as means ± SD or means ± SEM (*n* ≥ 3 for immunoblot quantifications and *n* ≥ 100 cells for confocal image quantifications). All data were parametric and the variance of the statistically compared groups was assumed as equal. A two‐tailed, unpaired Student’s *t*‐test was used for calculation of *P* values. Statistical significance was defined as *P* < 0.05.

## Supplementary Material

Supplemental Material

## Data Availability

The crystallographic data reported in this study have been deposited with the Protein Data Bank, accession numbers 6HOG, 6HOH, 6HOI, 6HOJ, 6HOK and 6HOL.
