# Supplementary material for: Members of the autophagy class III phosphatidylinositol 3-kinase complex I interact with GABARAP and GABARAPL1 via LIR motifs
Source: Autophagy. 2019 Mar 4;15(8):1333–55. doi: 10.1080/15548627.2019.1581009 (PMC6613885; doi:10.1080/15548627.2019.1581009)
Supplement: Supplemental Material [file kaup-15-08-1581009-s001.docx]

**Figure S1.** GST affinity-isolation assays with recombinant GST-Atg8 homologs and *in vitro*-translated ^35^S-labelled MYC-tagged PIK3R4 and PIK3C3. (**A**) Autoradiographs (AR, top panels) and Coomassie Brilliant Blue-stained immobilized GST or GST-fusion proteins (bottom panel) are shown. (**B**) Peptide arrays demonstrating increased binding of phosphomimetic E mutants at known phosphorylation sites preceding the LIR motifs of PIK3C3 and BECN1. The 24-mer peptides were spotted in triplicates and probed with 1 µg/ml of GST-Atg8 proteins for 2 h and binding was detected with anti-GST antibody.

**Figure S2.** Density maps of PIK3C3 LIR motifs. Electron density map of (**A**) WT PIK3C3 LIR chimera and (**B**) phosphomimetic PIK3C3^S249E^ LIR chimera. The Fo-Fc omit map of the LIR motifs is contoured at 3.0 σ. GABARAP is displayed in white cartoon and the LIR motif in cartoon and sticks.

**Figure S3.** Density maps of BECN1 LIR motifs. Electron density map of (**A**) WT BECN1 LIR peptide, (**B**) WT BECN1 LIR chimera and (**C**) phosphomimetic BECN1^S96E^ LIR chimera. The Fo-Fc omit map of the LIR motifs is contoured at 3.0 σ. GABARAP and GABARAPL1 are displayed in white cartoon and the LIR motif in cartoon and sticks.

**Figure S4.** Density map of ATG14 LIR motif. Electron density map of WT ATG14 LIR peptide. The Fo-Fc omit map of the LIR motifs is contoured at 3.0 σ. GABARAPL1 is displayed in white cartoon and the LIR motif in cartoon and sticks.

**Figure S5.** Variation in hydrophobic pocket 2 (HP2) may explain the specificity of PIK3C3, BECN1 and ATG14 LIRs for the GABARAP family. Structure superposition of (**A**) the wild type PIK3C3, (**B**) BECN1 and (**C**) ATG14 LIR motifs complexes with the published structure of LC3B (PDB ID:3VTU). PIK3C3, BECN1 and ATG14 LIR motifs structures are displayed as in figures 4A, 5A, 6A. LC3B is displayed in bright green cartoons. Only the LC3B residues H57, V58 and L63 discussed in the text are displayed in sticks.

**Figure S6.** Defects in autophagy caused by *ATG14* KO can be rescued with WT or LIR mutated Myc-ATG14. (**A**) Western blot experiments of indicated HCT116 cell lines confirming KO of endogenous *ATG14* and rescue with WT or LIR mutated MYC-ATG14. (**B-E**) Western blots of lysates from HCT116 WT cells (B), *ATG14* KO cells (C), KO cells rescued with MYC-ATG14 (D) and KO cells rescued with LIR-mutated MYC-ATG14 (E). Cells were treated as indicated before performing western blots with the indicated antibodies. The data shown are representative of 2 other experiments.

**Figure S7.** Colocalization of SQSTM1 with LAMP1 depends on ATG14 and is more efficient if ATG14 has a functional LIR motif. (**A**) HCT116 *ATG14* KO cells or cells reconstituted with WT or LIR-mutated ATG14 were treated as indicated, immunostained with SQSTM1 and LAMP1 antibodies, and analysed by confocal imaging. Scale bars: 10 μm. (**B**) Quantification of the colocalization analyzed in EV6A, represented as the percentage of SQSTM1-positive structures also positive for LAMP1. Data information: Means ± SEM; n ≥ 150 cells. Significant *P* values are indicated (Student´s two-tailed, unpaired *t*-test).
